# Supplementary material for: Induction of human pruriceptors from pluripotent stem cells via transcription factors
Source: Stem Cell Reports. 2026 Jul 9;21(8):103004. doi: 10.1016/j.stemcr.2026.103004 (PMC13476876; doi:10.1016/j.stemcr.2026.103004)
Supplement: Supplementary file 1 [file mmc1.pdf]

Stem Cell Reports, Volume *21*

Supplemental Information

Induction of human pruriceptors from pluripotent stem cells via transcription factors

Hisato Iriki, Ruiqi Hu, Xu Li, Erdene Baljinnyam, Carina Habich, Ichiro Imanishi, Loan N. Miller, Kavya Chegireddy, Laraib Iqbal Malik, Daniel Yassky, Raphael Kübler, Aaron Ver Heul, Kathleen M. Smith, Peter Reinhardt, Eric R. Goedken, Brian S. Kim, and Samuele G. Marro

Supplemental Material

Supplemental Figures

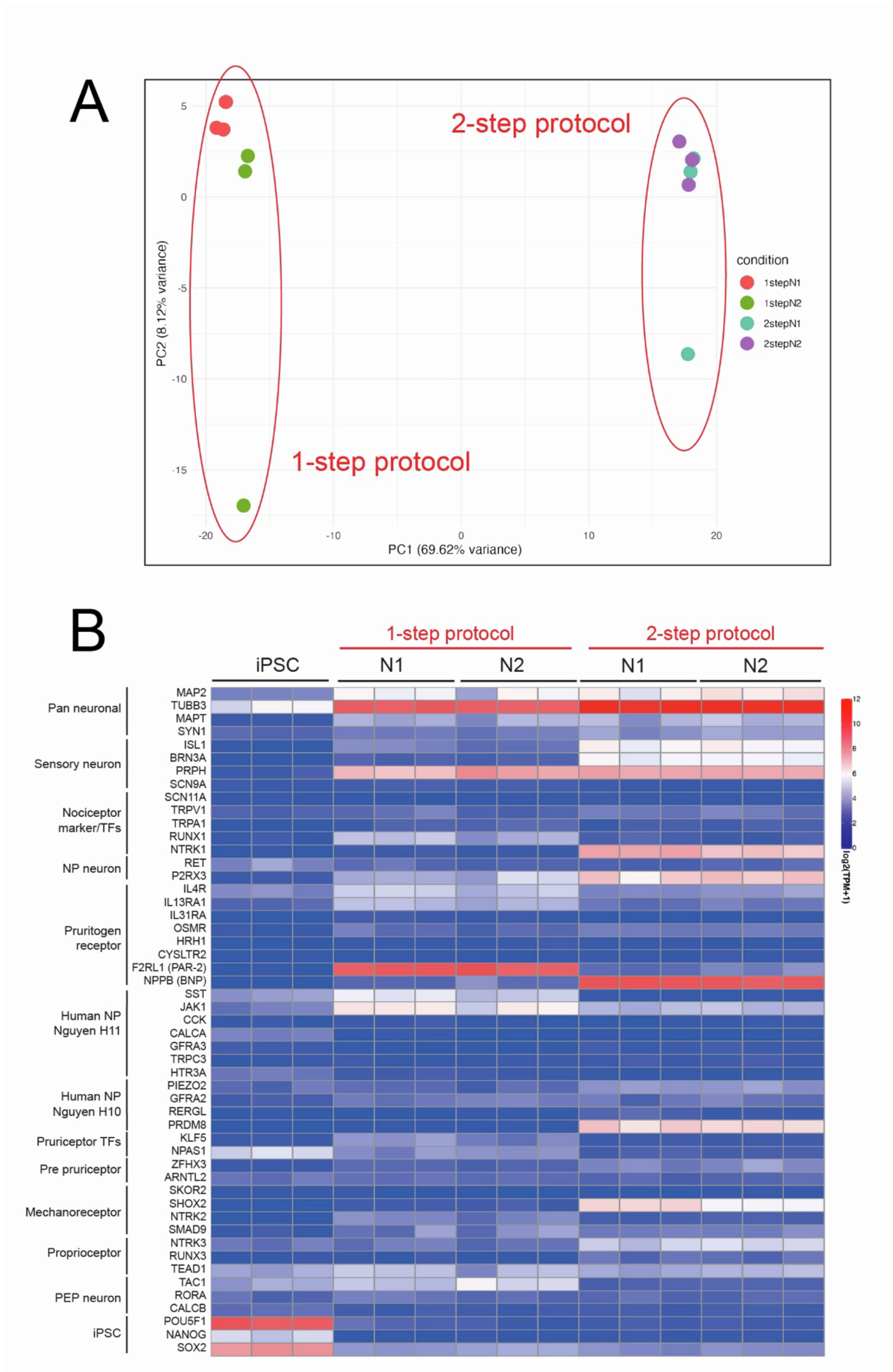

Figure S1. NGN1 expression in NCCs shows a tendency to enhance pruriceptor marker induction. Related to Figure 1.

(A) PCA of transcriptomic profiles. Each point represents a sample colored by condition: 1stepN1 (red), 1stepN2 (green), 2stepN1 (cyan), and 2stepN2 (purple). PC1 and PC2 explain 69.62% and 8.12% of the variance, respectively.

(B) Heatmap analysis of samples obtained on protocol day 21 using either the 1-step or 2-step protocol with NGN1 or NGN2 expression (1-stepN1, 1-stepN2, 2-stepN1, and 2-stepN2), along with iPSCs, showing the expression of the indicated markers. Transcript expression values (TPM) were log2-transformed, n=3. The analyzed data is same as the one analyzed in Figure 1C.

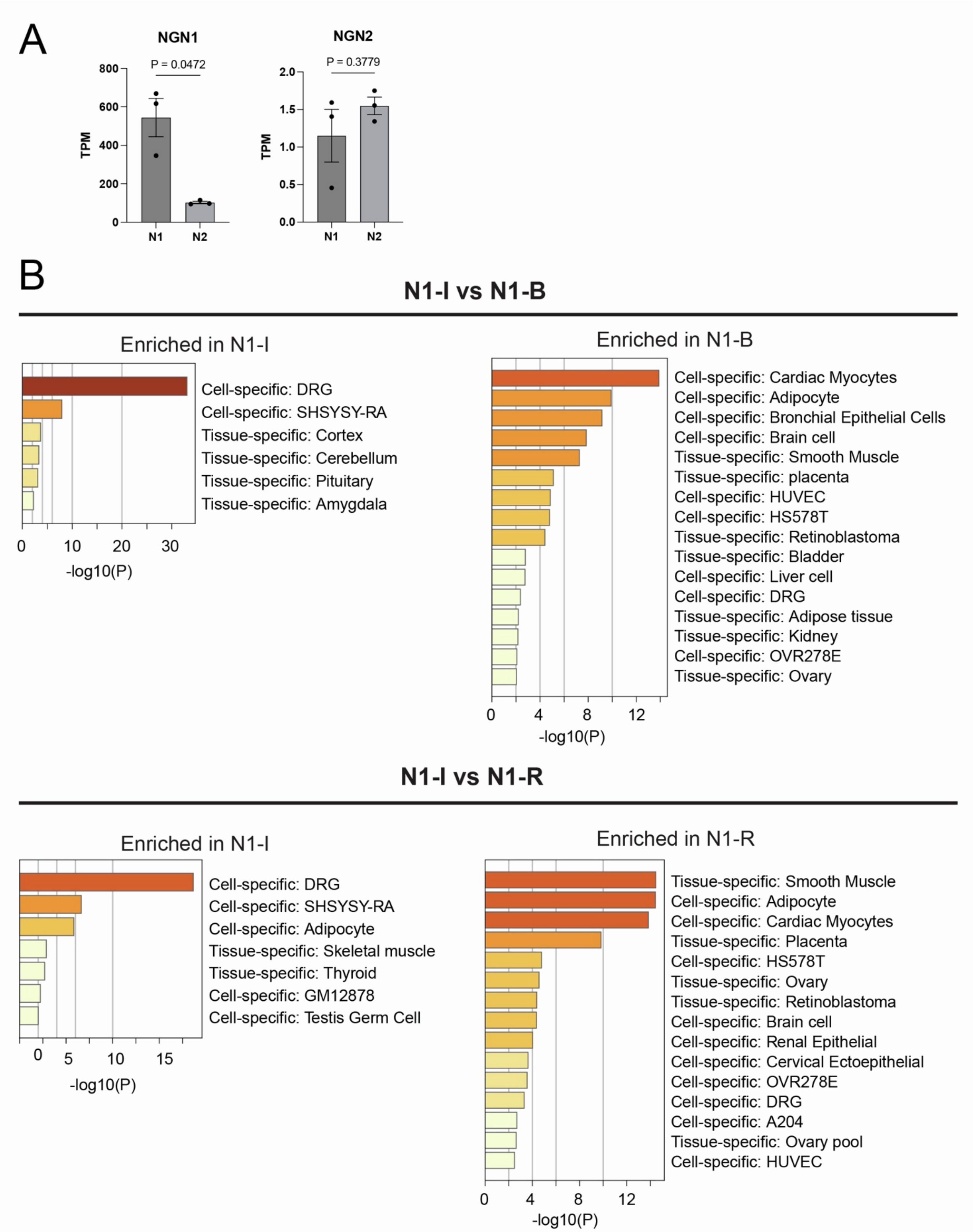
A
NGN1
P = 0.0472
TPM
800
600
400
200
0
N1
N2
NGN2
P = 0.3779
TPM
2.0
1.5
1.0
0.5
0.0
N1
N2
B
N1-I vs N1-B
Enriched in N1-I
Cell-specific: DRG
Cell-specific: SHSYSY-RA
Tissue-specific: Cortex
Tissue-specific: Cerebellum
Tissue-specific: Pituitary
Tissue-specific: Amygdala
0
10
20
30
-log10(P)
Enriched in N1-B
Cell-specific: Cardiac Myocytes
Cell-specific: Adipocyte
Cell-specific: Bronchial Epithelial Cells
Cell-specific: Brain cell
Tissue-specific: Smooth Muscle
Tissue-specific: placenta
Cell-specific: HUVEC
Cell-specific: HS578T
Tissue-specific: Retinoblastoma
Tissue-specific: Bladder
Cell-specific: Liver cell
Cell-specific: DRG
Tissue-specific: Adipose tissue
Tissue-specific: Kidney
Cell-specific: OVR278E
Tissue-specific: Ovary
0
4
8
12
-log10(P)
N1-I vs N1-R
Enriched in N1-I
Cell-specific: DRG
Cell-specific: SHSYSY-RA
Cell-specific: Adipocyte
Tissue-specific: Skeletal muscle
Tissue-specific: Thyroid
Cell-specific: GM12878
Cell-specific: Testis Germ Cell
0
5
10
15
-log10(P)
Enriched in N1-R
Tissue-specific: Smooth Muscle
Cell-specific: Adipocyte
Cell-specific: Cardiac Myocytes
Tissue-specific: Placenta
Cell-specific: HS578T
Tissue-specific: Ovary
Tissue-specific: Retinoblastoma
Cell-specific: Brain cell
Cell-specific: Renal Epithelial
Cell-specific: Cervical Ectoepithelial
Cell-specific: OVR278E
Cell-specific: DRG
Cell-specific: A204
Tissue-specific: Ovary pool
Cell-specific: HUVEC
0
4
8
12
-log10(P)

Figure S2. ISL1 contributes to differentiation into DRG neurons when co-expressed with NGN1. Related to Figure 2.

(A) Bar graphs showing TPM of NGN1 and NGN2 in N1 and N2. Each bar represents the mean ± SEM, and individual data points are shown. Statistical comparisons between the two groups were performed using an unpaired t-test with Welch's correction.

(B) Metascape tissue-specific enrichment of the top 300 DEGs: upper panels show 2-step N1-I vs N1-B and lower panels 2-step N1-I vs N1-R; within each comparison, left bars represent tissues/cell types enriched among genes up-regulated in N1-I and right bars those enriched in N1-B or N1-R, with bar length indicating $-\log_{10}$ P.

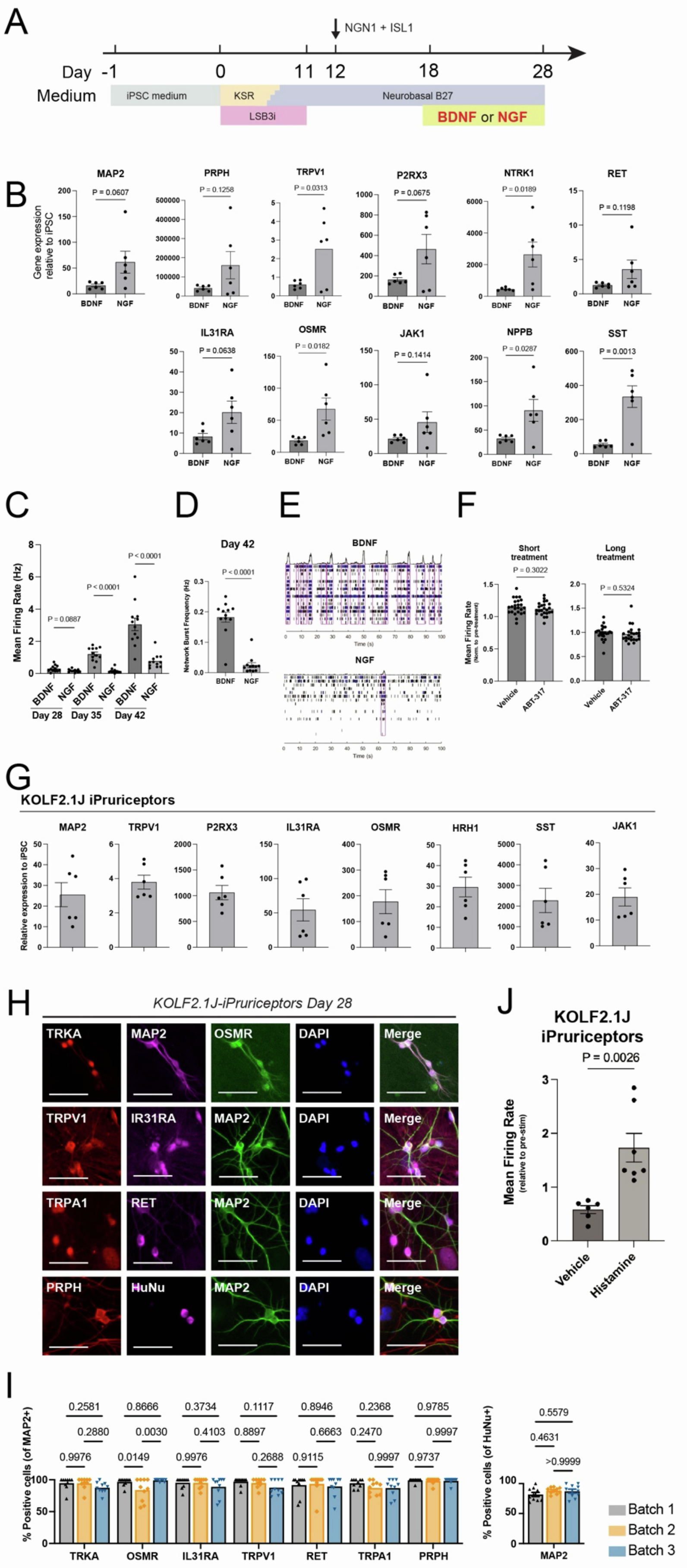

A
NGN1 + ISL1
Day -1 0 11 12 18 28
Medium
iPSC medium
KSR
Neurobasal B27
LSB3i
BDNF or NGF
B
Gene expression relative to iPSC
MAP2 P = 0.0607
PRPH P = 0.1258
TRPV1 P = 0.0313
P2RX3 P = 0.0675
NTRK1 P = 0.0189
RET P = 0.1198
IL31RA P = 0.0638
OSMR P = 0.0182
JAK1 P = 0.1414
NPPB P = 0.0287
SST P = 0.0013
BDNF NGF
C
Mean Firing Rate (Hz)
P = 0.0887
P < 0.0001
P < 0.0001
BDNF NGF BDNF NGF BDNF NGF
Day 28 Day 35 Day 42
D
Day 42
Network Burst Frequency (Hz)
P < 0.0001
BDNF NGF
E
BDNF
NGF
Time (s)
F
Short treatment
P = 0.3022
Long treatment
P = 0.5324
Mean Firing Rate (Norm. to pre-treatment)
Vehicle ABT-317
G
KOLF2.1J iPruriceptors
Relative expression to iPSC
MAP2 TRPV1 P2RX3 IL31RA OSMR HRH1 SST JAK1
H
KOLF2.1J-iPruriceptors Day 28
TRKA MAP2 OSMR DAPI Merge
TRPV1 IR31RA MAP2 DAPI Merge
TRPA1 RET MAP2 DAPI Merge
PRPH HuNu MAP2 DAPI Merge
J
KOLF2.1J iPruriceptors
P = 0.0026
Mean Firing Rate (relative to pre-stim)
Vehicle Histamine
I
% Positive cells (of MAP2+)
0.2581 0.8666 0.3734 0.1117 0.8946 0.2368 0.9785
0.2880 0.0030 0.4103 0.8897 0.6663 0.2470 0.9997
0.9976 0.0149 0.9976 0.2688 0.9115 0.9997 0.9737
TRKA OSMR IL31RA TRPV1 RET TRPA1 PRPH
% Positive cells (of HuNu+)
0.5579
0.4631
>0.9999
MAP2
Batch 1
Batch 2
Batch 3

Figure S4. iPruriceptors functionally respond to pruritogenic stimuli and JAK1 inhibition. Related to Figure 4.

(A) Schematic showing co-expression of NGN1 and ISL1 in NCCs induced by LSB3i via lentiviral transduction, followed by culture with BDNF or NGF from day 18 to day 28, the time point of quantitative PCR analysis.
(B) Quantitative PCR analysis of indicated markers on day 28 in the protocols under different neurotrophic conditions (BDNF vs NGF) after day 18. Relative expression levels were normalized to control iPSC levels. Data are presented as mean ± SEM, with individual data points representing independent biological replicates. Statistical comparisons were conducted using an unpaired t-test.
(C, D) MEA analysis showing maturation of neuronal activity over time under different neurotrophic conditions (BDNF vs NGF) after day 18. Mean firing rates were measured on days 28, 35, and 42, and network burst frequency was measured on day 42. The values represent the mean firing rate on (C) and network burst frequency on (D). Data are presented as mean ± SEM. Each dot represents one well. Representative data from three independent experiments (n = 12). Statistical comparisons between the two groups were performed using an unpaired t-test with Welch's correction.
(E) MEA raster plots showing neuronal spiking activity of neuronal activity with displaying network burst enclosed in purple box on day 42 under different neurotrophic conditions (BDNF vs NGF) after day 18. Each vertical line represents an action potential recorded from a single electrode.
(F) MEA analysis showing firing rates after short (30 sec) or long (60 min) treatment of JAK1 inhibitor, ABT-317, or vehicle treatment. Data are presented as mean ± SEM. Each dot represents one well. Pooled data from two independent experiments (n = 12 each).
(G) Quantitative PCR analysis of indicated markers on day 21 in the protocols using KOLF2.1J cells. Relative expression levels were normalized to control iPSC levels. Data are presented as mean ± SEM.
(H) Immunofluorescence staining of indicated markers on the induced neurons under iPruriceptor protocol from KOLF2.1J on day 28. Scale bars indicate 50μm.

(I) Bar graph showing the proportion of induced neurons under iPruriceptor protocol expressing the indicated markers on day 28. Data are presented as mean ± SEM across three independent batches. Each dot represents one field of view. Statistical comparisons between the batches were performed using a two-way ANOVA test.

(J) MEA analysis showing neuronal responses to histamine in induced neurons, using KOLF2.1J cell line, on day 42 from the iPruriceptor protocol maintained with NGF after day 18. Representative data from two independent experiments ($n = 6$ or 7). Values represent mean firing rates normalized to the average baseline across wells per condition. Each dot represents one well. Statistical comparisons between the two groups were performed using an unpaired t-test with Welch's correction.

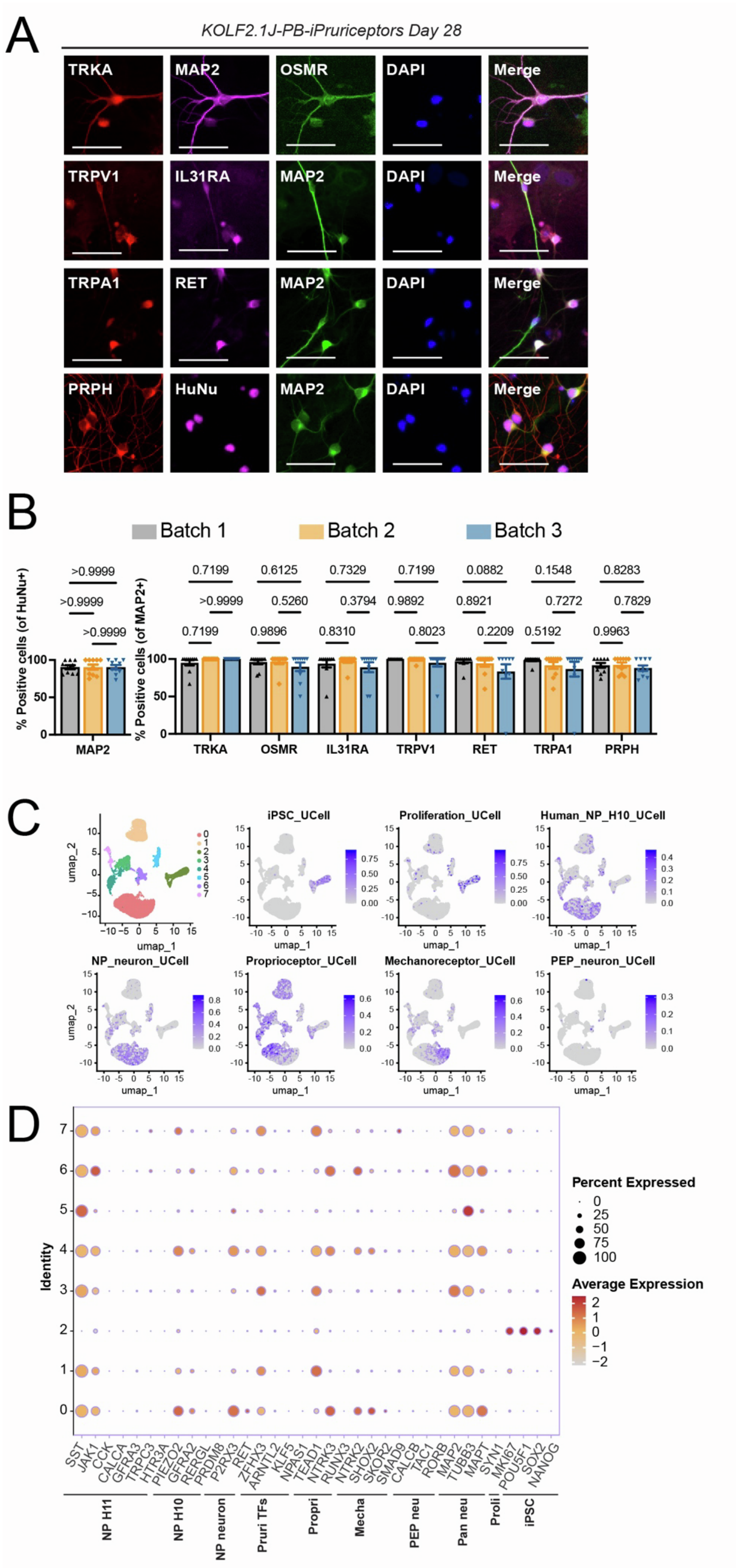
A
KOLF2.1J-PB-iPruriceptors Day 28
TRKA
MAP2
OSMR
DAPI
Merge
TRPV1
IL31RA
TRPA1
RET
PRPH
HuNu
B
Batch 1
Batch 2
Batch 3
% Positive cells (of HuNu+)
% Positive cells (of MAP2+)
MAP2
TRKA
OSMR
IL31RA
TRPV1
RET
TRPA1
PRPH
C
iPSC_UCell
Proliferation_UCell
Human_NP_H10_UCell
NP_neuron_UCell
Proprioceptor_UCell
Mechanoreceptor_UCell
PEP_neuron_UCell
umap_1
umap_2
D
Identity
Percent Expressed
Average Expression
SST JAK1 CCK CALCA GFRA3 TRPC3 HTR3A PIEZO2 GFRA2 RERGL PRDM8 P2RX3 RET ZFHX3 ARNTL2 KLF5 NPAS1 TEAD1 NTRK3 RUNX3 NTRK2 SHOX2 SKOR2 SMAD9 CALCB TAC1 RORB MAP2 TUBB3 MAPT SYN1 MKI67 POU5F1 SOX2 NANOG
NP H11
NP H10
NP neuron
Pruri TFs
Propri
Mecha
PEP neu
Pan neu
Proli
iPSC

Figure S5. iPruriceptors generated with PiggyBac system reproduce robust functional pruriceptor induction. Related to Figure 5.

(A) Immunofluorescence staining of indicated markers on the induced neurons under iPruriceptor protocol with PiggyBac on day 28 from KOLF2.1J. Scale bars indicate 50µm. (B) Bar graph showing the proportion of induced neurons under iPruriceptor protocol with PiggyBac expressing the indicated markers on day 28 from KOLF2.1J. Data are presented as mean ± SEM across three independent batches. Each dot represents one field of view. Statistical comparisons between the batches were performed using a two-way ANOVA test.

(C) UMAP visualization of snRNA-seq clusters with overlaid UCell scores for curated gene signatures. These scores reveal enrichment of neuronal subtype signatures and pluripotency signatures in iPruriceptor and iPSC.

(D) DotPlot showing expression of representative marker genes across clusters. Dot size indicates the percentage of nuclei expressing each gene, and color intensity represents average expression level, supporting the assignment of pruriceptor subtypes and differentiation stage identities.

Supplemental Tables

Table S1. Differentially expressed genes (DEGs) identified between the N1/N2/N1-I group and the N1-B/N1-R group generated using the 2-step differentiation protocol.

Table S2. Differentially expressed genes (DEGs) identified between the N1-I condition and the N1-B or N1-R conditions generated using the 2-step differentiation protocol.
