# Supplementary material for: Induction of human pruriceptors from pluripotent stem cells via transcription factors
Source: Stem Cell Reports. 2026 Jul 9;21(8):103004. doi: 10.1016/j.stemcr.2026.103004 (PMC13476876; doi:10.1016/j.stemcr.2026.103004)
Supplement: Supplementary file 2 [file mmc2.pdf]

STEM CELL REPORTS
Article

OPEN ACCESS

Induction of human pruriceptors from pluripotent stem cells via transcription factors

Hisato Iriki,[1,2,3,11] Ruiqi Hu,[4,5,11] Xu Li,[1,2,3] Erdene Baljinnyam,[5] Carina Habich,[6] Ichiro Imanishi,[1] Loan N. Miller,[7] Kavya Chegireddy,[8] Laraib Iqbal Malik,[8] Daniel Yassky,[1] Raphael Kübler,[4] Aaron Ver Heul,[9] Kathleen M. Smith,[8] Peter Reinhardt,[6] Eric R. Goedken,[8] Brian S. Kim,[1,2,3,4,10,12,13] and Samuele G. Marro[4,5,12,13,14,*]

[1]Kimberly and Eric J. Waldman Department of Dermatology, Icahn School of Medicine at Mount Sinai, New York, NY, USA
[2]Mark Lebwohl Center for Neuroinflammation and Sensation, Icahn School of Medicine at Mount Sinai, New York, NY, USA
[3]Marc and Jennifer Lipschultz Precision Immunology Institute, Icahn School of Medicine at Mount Sinai, New York, NY, USA
[4]Nash Family Department of Neuroscience, Friedman Brain Institute, Icahn School of Medicine at Mount Sinai, New York, NY, USA
[5]Department of Stem Cell Biology and Regenerative Medicine, Icahn School of Medicine at Mount Sinai, New York, NY, USA
[6]AbbVie Deutschland GmbH & Co KG, Neuroscience Discovery, Knollstrasse, Ludwigshafen am Rhein, Germany
[7]AbbVie Inc., North Chicago, IL, USA
[8]AbbVie Cambridge Research Center, Cambridge, MA, USA
[9]Sanofi, Immunology and Inflammation Therapeutic Area, Cambridge, MA, USA
[10]Allen Discovery Center for Neuroimmune Interactions, Icahn School of Medicine at Mount Sinai, New York, NY, USA
[11]These authors contributed equally
[12]These authors contributed equally
[13]Senior author
[14]Lead contact
*Correspondence: samuele.marro@mssm.edu
https://doi.org/10.1016/j.stemcr.2026.103004

SUMMARY

Pruriception is crucial for defense against external stimuli but can lead to chronic pruritus, a debilitating condition affecting millions worldwide. Our understanding of the cellular and molecular mechanisms behind the sensation of itch has been hindered by the lack of functional human models. Here, we address this limitation by developing a protocol to generate induced pruriceptors (iPruriceptors) from human-induced pluripotent stem cells (iPSCs). We compared two differentiation approaches: a direct method via forced expression of transcription factors (TFs) in iPSCs and a 2-step process through expression of TFs in iPSC-derived neural crest cells (NCCs). The 2-step protocol proved superior in inducing a transcriptional program enriched for features of human pruriceptors. Our optimized protocol employs forced expression of NGN1 and ISL1 to drive differentiation from NCCs into pruriceptors, enhancing the expression of known pruritogen receptors such as *IL31RA*, which pairs with *OSMR*, and *HRH1*. The induction of this transcriptional program leads to functional maturation of iPruriceptors. Accordingly, iPruriceptors exhibit robust responses to itch stimuli and *in vivo*-like itch pharmacology such as treatment with ABT-317, a JAK1 inhibitor tool compound, similar to those targeting intensive pruritus in atopic dermatitis (AD). Importantly, iPruriceptors can be generated without viral vectors or safe-harbor gene editing, using a PiggyBac-based transfection method that simplifies scalability. Our protocol offers a robust platform for investigating itch biology, modeling chronic pruritus, and enabling high-throughput screening for therapeutic target discovery.

INTRODUCTION

Pruriception, the perception of itch, helps the body defend against parasites and environmental irritants by provoking the desire to scratch. However, excessive and prolonged itch can lead to chronic pruritus, defined as itch lasting more than six weeks. Chronic pruritus affects over seven million people annually in the United States alone and can arise from multiple underlying causes, including immunological factors, systemic diseases, neuropathic origins, infections, drug-induced reactions, and/or idiopathically as in chronic pruritus of unknown origin (CPUO), among others (Butler et al., 2024; Kim et al., 2019).

The molecular mechanisms of itch begin with a stimulus in the skin that triggers local cells to produce cytokines and mediators or through direct activity on itch-sensory neurons called pruriceptors. Stimuli that elicit itch by acting directly on key molecular receptors on pruriceptors are referred to as pruritogens. Once activated, these receptors initiate a complex signaling cascade (Chen et al., 2020), leading to neuronal depolarization, action potential generation, and neurotransmitter release. This signal is then transmitted along the spinothalamic tract to the brainstem, where it is processed and relayed to various brain regions, ultimately producing the urge to scratch (Chen et al., 2020; Wang et al., 2021).

A well-known pruritogen is histamine, which mediates acute itch by activating the HRH1 receptor (histaminergic pathway) upon release from mast cells (Yosipovitch et al., 2018). Other mediators include IL-4, IL-13, and IL-31, type 2 cytokines produced by lymphocytes and mast cells, which activate their receptors for stimulating non-histaminergic

© 2026 The Author(s).
Published by Elsevier Inc. on behalf of International Society for Stem Cell Research.
This is an open access article under the CC BY license (http://creativecommons.org/licenses/by/4.0/).

itch pathways. Other canonical markers of pruriceptors include PAR-2, CysLTR2, somatostatin (SST), and B-type natriuretic peptide (BNP). Among these, BNP serves as a key neurotransmitter that mediates itch transmission to the spinal cord by activating NPR-A receptors. Notably, mice lacking BNP or NPR-A exhibit reduced responses to multiple itch stimuli, highlighting its crucial role in itch signaling (Meng et al., 2021; Mishra and Hoon, 2013). Common itch-associated cytokine receptors signal through the Janus kinase/signal transducer and activator of transcription (JAK/STAT) (Oetjen et al., 2017). Patients with recalcitrant chronic itch that have failed other immunosuppressive therapies have shown marked improvement when treated with Janus kinase 1 (JAK1)-selective inhibitors (Huang et al., 2024; Jeon et al., 2022; Kim et al., 2020, 2021). Indeed, JAK1 is highly expressed in itch neurons in human dorsal root ganglia (DRG) (Nguyen et al., 2021). Beyond canonical markers, recent single cell-multiomic studies of mouse and human DRG have expanded and reclassified neuronal subtypes across species. Interestingly, pruriceptors are among the most divergent groups between humans and mice. One study identified new classes of human pruriceptors named H10 and H11, which share some features with new classes of mouse pruriceptors named NP1 and NP3 (Nguyen et al., 2021; Usoskin et al., 2015). However, a unique feature of human pruriceptors is that they exhibit many more non-pruriceptive receptors, suggesting their capacity for functional polymodality (Jung et al., 2023). Thus, the ability to functionally evaluate human sensory neurons would greatly advance clinical development of novel anti-pruritic therapies.

Developmentally, pruriceptors are a subclass of nociceptors, specifically belonging to the non-peptidergic (NP) class, in contrast to the peptidergic (PEP) class. Like other nociceptors, pruriceptors originate from neural crest cells (NCCs) or, in the case of trigeminal nociceptors, from the head placode. Nociceptors are characterized by the expression of TrkA (tropomyosin receptor kinase A), the high-affinity receptor for nerve growth factor (NGF) and are generated during the last wave of sensory neuron development (Ma et al., 1999; Sommer et al., 1996; Zirlinger et al., 2002). Proprioceptors are the earliest to develop and express TrkC, the receptor for neurotrophin-3 (NT-3). Mechanoreceptors follow, expressing TrkB, the receptor for brain-derived neurotrophic factor (BDNF). The first two waves of sensory neuron development (proprioceptors and mechanoreceptors) are driven by Neurogenin-2 (NGN2), while the third wave (nociceptors including pruriceptors) is controlled by Neurogenin-1 (NGN1) (Marmigere and Ernfors, 2007).

Numerous protocols have successfully recapitulated this complex developmental cascade *in vitro*, enabling differentiation of human-induced pluripotent stem cells (iPSCs) into sensory neurons using either extrinsic factors, forced expression of lineage-specifying transcription factors (TFs), or a combination of both. For instance, iPSCs have been directed toward NCCs and subsequently induced to adopt a sensory neuron fate through the forced expression of TFs such as NGN1 or NGN2. The resulting sensory neurons exhibit characteristics of general nociceptors (Holzer et al., 2022; Lallemend and Ernfors, 2012; Marmigere and Ernfors, 2007; Nickolls et al., 2020; Plumbly et al., 2022; Schrenk-Siemens et al., 2022). However, it has been reported that there is no difference in nociceptor induction efficiency between NGN1 and NGN2, at least in forced expression in fibroblasts (Blanchard et al., 2015), and the significance of NGN1 over NGN2 in NCC and the ideal use of these TFs have not been clarified completely. In addition, it is known that other TFs are also expressed in discrete stages during nociceptors differentiation. How they work in conjunction with NGN1 and NGN2 in the differentiation of iPSCs is largely unknown.

To date, no protocol has been established for specifically generating pruriceptors from iPSCs through the expression of lineage-specifying TFs. Here, we describe a novel protocol that generates pruriceptor-enriched human sensory neurons from iPSCs through the forced expression of lineage-specifying TFs. The resulting cells (iPruriceptors) express markers and receptors for pruritogens identified in primary DRG single-cell transcriptomic studies. Using a stepwise protocol that mimics *in vivo* sensory neuron differentiation, we confirmed their responsiveness to itch stimuli and the ability of a JAK1-selective inhibitor to disrupt their activity in line with the known anti-pruritic effects of clinical JAK1 inhibition. This approach provides a reliable, reproducible and scalable method for generating human pruriceptors, offering new insights into pruriception and potential therapeutic applications for chronic itch disorders.

RESULTS

Enhanced pruriceptor fate induction by NGN1 overexpression in NCCs

Protocols for the differentiation of iPSCs to neural lineage based on extrinsic factors faithfully reproduce the stepwise processes of neurogenesis but are known to be inefficient and lengthy (Chambers et al., 2009; Mica et al., 2013). We therefore turned to the widely used ectopic expression of the master regulator of neurogenesis, NGN2, to bypass the progenitor state and rapidly differentiate iPSCs to induced neurons (iN) with minimal line-to-line variability (Zhang et al., 2013). To optimize the specification of iN cells toward pruriceptors, we focused on a panel of markers typically expressed by primary human pruriceptors as

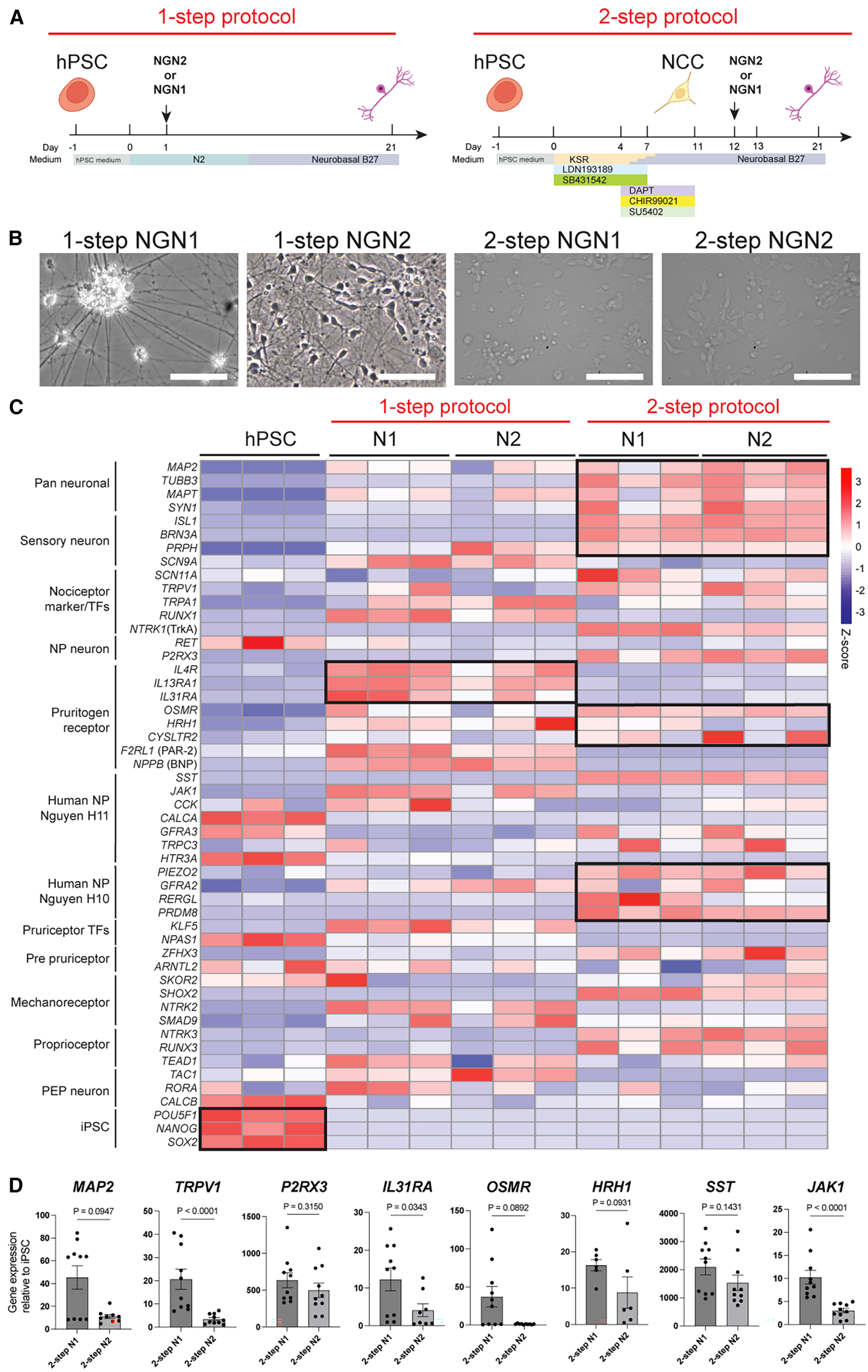

(legend on next page)

defined by the new classes H10 and H11 (Nguyen et al., 2021). In parallel to NGN2, we also tested NGN1 because of its role in the neurogenesis of pruriceptors (Ma et al., 1999; Sommer et al., 1996; Zirlinger et al., 2002). NGN1 has been used to generate motor neurons from iPSCs (Goparaju et al., 2017), mixed population of sensory neurons from fibroblasts (Blanchard et al., 2015) and iPSCs (Holzer et al., 2022). We therefore asked whether NGN2 and NGN1 have different effects when overexpressed in iPSC (1-step protocol) or in iPSC-derived NCCs (2-step protocol). For the 1-step protocol, we used lentiviral transduction for constitutive expression of rtTA (Urlinger et al., 2000) and tetracycline-inducible expression driven by a tetracycline (Tet) inducible promoter to express either NGN1 or NGN2 in iPSCs, followed by doxycycline induction on day 1 and positive selection with puromycin on day 2. For the 2-step protocol, we first differentiated iPSCs into NCCs using the LSB3i protocol previously reported (Chambers et al., 2012). At day 11, we transduced the NCCs with the same lentiviral constructs to express either NGN1 or NGN2, followed by doxycycline induction on day 12 and positive selection with puromycin (Puro) on day 13 (Figure 1A). At day 21 both protocols produced bipolar morphology typical of iN cells (Figure 1B). We then analyzed the transcriptomic profile of the resulting iN cells using RNA sequencing (RNA-seq) and confirmed that pluripotency markers, such as *POU5F1* and *NANOG* were efficiently downregulated in both protocols (Figures 1C and S1B). At the same time, canonical neuronal lineage markers, including *MAPT*, *MAP2*, *TUBB3*, and *SYN1*, were upregulated, consistent with robust neuronal specification. Interestingly, the 2-step protocol more effectively induced pan-neuronal markers such as *MAP2* and *MAPT*, indicating a more robust promotion of neuronal differentiation. Notably, the choice of protocol (1-step vs. 2-step) had a greater impact on differentiation outcomes than the identity of the overexpressed TF as shown by the principal component analysis (Figure S1A). Within the 1-step protocol, however, NGN1 induced higher levels of nociceptor-associated genes, including *RUNX1* and *TRPV1*, relative to the NGN2 protocol. Similarly, NGN1 outperformed NGN2 in promoting the expression of pruritogen receptors and associated signaling components, such as *IL13RA1*, *IL31RA*, *OSMR*, *HRH1*, and *JAK1* (Figures 1C and S1B). For H10 markers, the 2-step protocol—particularly in combination with NGN1—also showed a distinct advantage. Similarly, the expression of *HRH1*, one of the most representative pruritogen receptors, was higher in 2-step NGN1 than in 2-step NGN2. In a separate differentiation batch, we performed quantitative real-time PCR (real-time qPCR) to directly compare the efficiency of NGN1 and NGN2 in the two-step protocol for inducing the expression of key genes, including *IL31RA*, *OSMR*, and *JAK1*. Overall, NGN1 demonstrated superior performance (Figure 1D).

Together, these results suggest that NGN1 has a distinct capacity to initiate specific transcriptional programs compared to NGN2 and that the execution of these programs is influenced by both the cellular context in which NGN1 is overexpressed and the extrinsic signals present during induction. Given the superior efficiency of the 2-step protocol in promoting neuronal gene expression and suppressing the NCC program, along with the enhanced induction of pruriceptor markers, we selected the 2-step NGN1 protocol for subsequent refinement.

ISL1 contributes to DRG cell fate specification when co-expressed with NGN1

To further refine a protocol for the generation of iN that faithfully represent human pruriceptors, we added additional TFs that are involved in the second wave of neurogenesis, namely ISL1, BRN3A, and RUNX1 to the NGN1-2-step overexpression protocol (Lallemend and Ernfors, 2012; Marmigere and Ernfors, 2007) (Figures 2A and 2B). These refined protocols—named 2-step N1-I, 2-step N1-B, and 2-step N1-R—produced iN cells with the same apparent efficiency of the 2-step N1 protocol with no effect on morphology. We then analyzed the effect of the added TFs on the global transcription profile of the iN. Not surprisingly, we observed some level of cross-induction between the TFs overexpressed in the different protocols, for instance, in the N1 protocol NGN1 induced the

Figure 1. NGN1 expression in NCCs shows a tendency to enhance pruriceptor marker induction

(A) Schematic of NGN1 and NGN2 expression in iPSCs (1-step protocol) and NCCs induced by LSB3i (2-step protocol) using a lentiviral system.

(B) Representative images showing neuronal morphology in 1-step NGN1, 1-step NGN2, 2-step NGN1, 2-step NGN2 on day 21. Scale bars indicate 100 μm.

(C) Heatmap analysis of samples obtained on protocol day 21 using either the 1-step or 2-step protocol with NGN1 or NGN2 expression (1-stepN1, 1-stepN2, 2-stepN1, and 2-stepN2), along with iPSCs, showing the expression of the indicated markers. Transcript expression values (TPM) were log2-transformed, and *Z* scores were calculated, $n = 3$.

(D) Quantitative PCR analysis of indicated markers in 2-step N1 and 2-step N2 protocol on day 21. Relative expression levels were normalized to control iPSC levels. Data are presented as mean ± SEM, with individual data points representing independent biological replicates. Statistical comparisons were conducted using an unpaired *t* test ($n = 3$). Also see Figure S1.

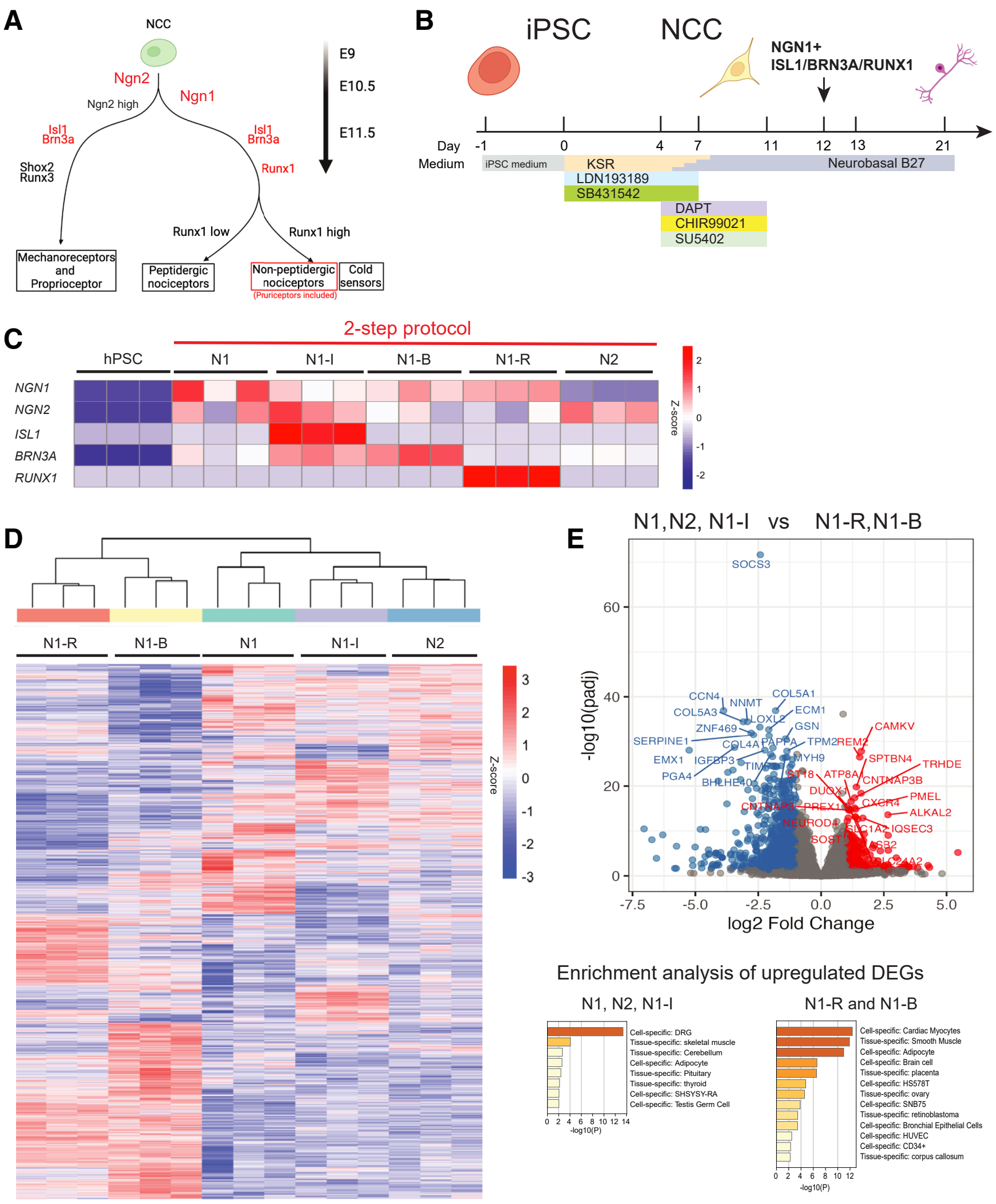

Figure 2. ISL1 contributes to differentiation into DRG neurons when co-expressed with NGN1

(A) Schematic of peripheral nerve developmental cascade in mice highlighting the role of NGN1, ISL1, BRN3A, and RUNX1 in NP-nociceptor differentiation.

(B) Schematic of co-expression of NGN1 with ISL1, BRN3A, and RUNX1 expression in NCCs induced by LSB3i using a lentiviral system.

(C) Heatmap analysis of samples obtained on protocol day 21 using 2-step N1, N1-I, N1-B, N1-R, and N2, along with iPSCs, showing the expression of the introduced TFs; NGN1, NGN2, ISL1, BRN3A, and RUNX1. Transcript expression values (TPM) were log10-transformed, and *Z* scores were calculated.

(D) Heatmap analysis and hierarchical clustering using the expression of DEGs on the samples from 2-step N1, N1-I, N1-B, N1-R, and N2.

(E) Volcano plot displaying differentially expressed genes (DEGs) identified by comparing the N1/N2/N1-I group with the N1-R/N1-B group (all samples derived from the 2-step protocol). The *x* axis shows log2 fold change and the *y* axis shows −log10 (adjusted *p* value). Genes with an adjusted *p* value <0.05, calculated with DESeq2, were considered significantly up- or down-regulated. The bar charts below summarize Metascape enrichment (−$\log_{10}$ P) of the top 300 upregulated genes: left, tissues/cell types over-represented in N1/N2/N1-I; right, those over-represented in N1-R/N1-B. Also see Figure S2.

expression of *NEUROG2* (*NGN2* ; to the same levels found in the N2 protocol), but in the N2 protocol, NGN2 only weakly induced *NEUROG1* (*NGN1)* (Figure S2A). Also, ISL1 expression in the context of N1-I protocol synergized with NGN1 to further induce *NGN2* expression and the expression of *POU4F1* (*BRN3A)*. On the contrary, no cross-induction was observed in the N1-B or N1-R protocols (Figure 2C). On a global scale, the transcriptional profile of the iN was majorly altered by the addition of BRN3A or RUNX1, as indicated by the unsupervised hierarchical clustering of variance-stabilized counts (Pearson-correlation distance, Ward's linkage) of the N1-B and N1-R in a separate group from the N1, N2, and N1-I (Figure 2D).

We then performed differentially expressed gene (DEG) analysis of these two groups and observed that only the second group (N1, N2, and N1-I) expressed genes associated to general sensory neuron identity, such as *ALKAL2* and *REM2* (Defaye et al., 2022; Liput et al., 2016) (Figures 2E and S2B; Table S1). On the other hand, the first group (N1-B and N1-R) had robust expression of non-peripheral nervous system (PNS) markers (Figure 2E; Table S1). Accordingly, N1-I showed high expression of genes related to PNS and DRG neurons when it was compared with N1-B or N1-R (Figure S2B; Table S2). Together, these results suggest that ISL1—but not BRN3A and RUNX1—when co-expressed with NGN1, contributes to the activation of a transcriptional program resembling that of DRG neurons.

In addition to enhancing a DRG-like transcriptional program, the combination of NGN1 and ISL1 also promoted the expression of pruritogen receptors. Compared to NGN1 alone, N1-I iN cells exhibited stronger expression of PNS markers, such as peripherin (*PRPH*), nociceptor markers (*RET* and *P2RX3*), pruritogen receptors and

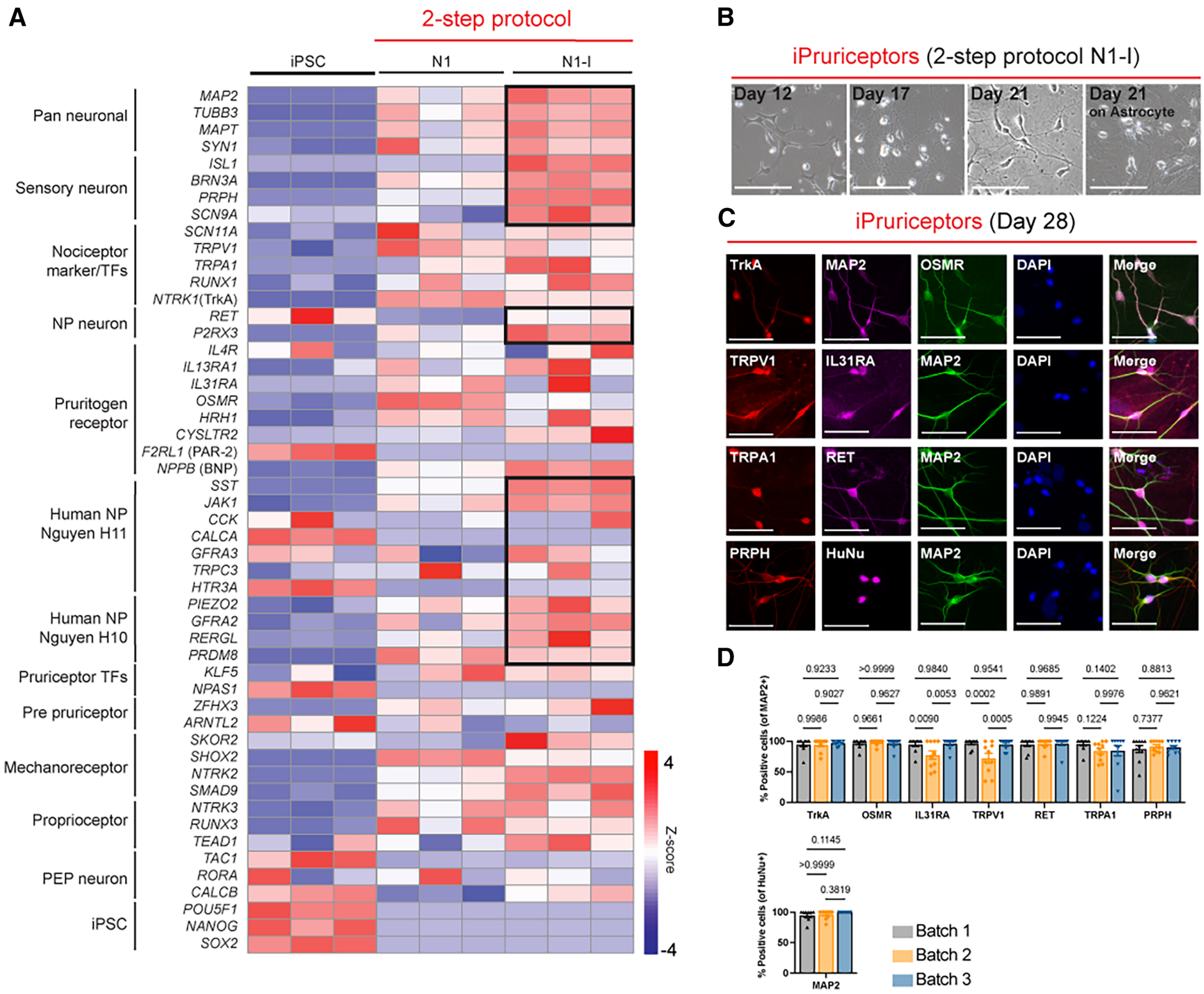

Figure 3. Co-expression of NGN1 and ISL1 induce pruriceptor marker expression

(A) Heatmap analysis on the samples from 2-step N1 and N1-I, showing the expression of indicated markers. Transcript expression values (TPM) were log10-transformed, and Z scores were calculated.

(B) Representative images showing neuronal morphology at different stages of differentiation under the 2-step N1-I, namely iPruriceptor, protocol on day 12, 17, and 21. Scale bars indicate 100 μm.

(C) Immunofluorescence staining of indicated markers on the induced neurons under iPruriceptor protocol on day 28. Scale bars indicate 50 μm.

(D) Bar graph showing the proportion of induced neurons under iPruriceptor protocol expressing the indicated markers on day 28. Data are presented as mean ± SEM across three independent batches. Each dot represents one field of view. Statistical comparisons between the batches were performed using a two-way ANOVA test.

associated signaling proteins (e.g., *JAK1*), as well as markers of the H10 and H11 neuronal subclasses (Figure 3A).

Based on these findings, we conclude that co-expression of NGN1 and ISL1 in NCCs drives pruriceptor specification. We therefore designated these cells as "iPruriceptors", reflecting their rapid neuronal differentiation (Figure 3B), evident by day 12, and their robust expression of pruriceptor markers—including TrkA, OSMR, IL31RA, TRPV1, RET, TRPA1, and PRPH—by day 28, as confirmed by immunocytochemistry (Figure 3C). To assess reproducibility, we quantified MAP2$^+$ neuronal cells and their expression of pruriceptor markers across three independent differentiation batches. In all cases, >88% of neurons expressed the markers, and neurons represented >97% of HuNu$^+$ human cells. When defined as MAP2 and TrkA double-positive cells, iPruriceptors accounted for more than 93% of the produced cells. Neuronal yield was consistent across batches, although IL31RA and TRPV1 expression showed modest but statistically significant differences in batch 2 relative to batches 1 and 3 (Figure 3D). Expression of other markers remained comparable across batches. Overall, these results support the robustness of the differentiation

protocol and the consistent generation of neurons highly enriched for pruriceptor-associated markers.

iPruriceptors selectively respond to pruritogenic stimuli and JAK1 inhibition

Next, we sought to determine whether iPruriceptors are electrophysiologically active and capable of responding to specific pruritogenic stimuli, such as histamine and IL-31, as well as to general, non-pruritogenic agents like capsaicin which signals via TRPV1. To promote their functional maturation, we co-cultured iN cells with primary mouse glial cells starting on day 18, following our previously established iN protocols (Marro et al., 2019; Pang et al., 2011; Zhang et al., 2013). The culture medium of iN cells is typically supplemented with BDNF, the primary ligand of TrkB, a classical marker of mechanoreceptors (Lu et al., 2024). We therefore tested the effect of supplementing the maturation medium with NGF, the ligand for TrkA, a key marker of nociceptors, and asked whether NGF—administered during the maturation phase rather than during initial induction—could enhance the electrophysiological properties of iPruriceptors (Figure S4A).

Importantly, because this supplementation occurred after the transcriptional profile of the cells was presumably already established, we predicted a negligible impact on overall cell fate. Nevertheless, to exclude this possibility, we assessed the expression of key pruriceptor marker genes under both conditions and found that all expected markers remained robustly expressed—with some even upregulated in NGF-supplemented cultures—suggesting a robust differentiation into pruriceptors (Figure S4B). We next compared the two maturation conditions—BDNF vs. NGF—at the functional level using multielectrode array (MEA) recordings (Figures 4A and 4B). iPruriceptors matured in NGF-containing media exhibited a lower basal activity compared to those matured in BDNF, as measured by mean firing rate (MFR) (Figures 4C and S4C). BDNF cultures also displayed more frequent network bursts (Figures S4D and S4E), indicative of synchronized firing often observed in glutamatergic CNS neurons.

To evaluate stimulus-induced activity, we exposed iPruriceptors to histamine and IL-31. iN cells matured in BDNF-containing media showed no change in MFR following stimulation (Figure 4D). In contrast, induced neurons derived with the lentiviral-based protocol and cultured with NGF exhibited a significant increase in MFR in response to histamine, IL-31, and IL-13 (Figure 4E). Capsaicin did not significantly alter MFR under these MEA recording conditions (Figure 4F). MFRs were quantified relative to each well's pre-stimulation baseline ("relative to pre-stim"), ensuring that stimulus-induced changes reflect within-well normalization rather than absolute differences in baseline activity. Finally, we tested whether the IL-31 and IL-13 responses were mediated by JAK1 signaling using the JAK1 inhibitor tool compound ABT-317 (Ge et al., 2020; Todorovic et al., 2022; Twomey et al., 2024). Inhibition of neuronal JAK1 pathways has also been shown to ameliorate pruritus (Oetjen et al., 2017). Remarkably, this treatment fully suppressed the cytokine driven MEA response, indicating that iPruriceptors respond specifically via the JAK1-dependent pathway (Figure 4G), consistent with the expected anti-pruritogenic properties. Importantly, treatment with JAK1 inhibitor alone, in the absence of IL-31 and IL-13, did not significantly alter MFR compared to vehicle controls (Figure S4F). To extend the validation of these results and test the robustness of the protocol across a broader range of iPSC lines, we generated iPruriceptors from one additional iPSC line, the KOLF2.1J (Pantazis et al., 2022). KOLF2.1J produced iPruriceptors with similar efficiency of our reference line (WTC11), showing the pruriceptor marker expression on day 21 by qPCR and immunohistochemically on day 28 (Figures S4G–S4I). Additionally, the resulting iPruriceptors were functionally active, as shown by their response to histamine (Figure S4J). These functional data support the utility of iPruriceptors as a relevant *in vitro* platform for translational studies of pruritus.

Finally, to enhance the scalability of the iPruriceptors protocol across multiple iPSC lines, we sought alternatives to lentiviral delivery of NGN1 and ISL1, a common bottleneck in iN cell production. As an alternative, we employed the PiggyBac (PB) transposon system, previously used for iN cell generation (Gu et al., 2022). We constructed a polycistronic PB vector containing (1) the reverse transactivator rtTA under the control of CAG promoter; (2) a puromycin resistance gene and a nuclear-localized BFP under the control of EF1α promoter; (3) a ubiquitous chromatin opening element (UCOE) to attenuate transgene silencing in iPSCs and their differentiated cells (Durens et al., 2024; Muller-Kuller et al., 2015); and (4) a bidirectional doxycycline-inducible TRE3G BI promoter to drive co-expression of NGN1 and ISL1 (Figure 5A). Following PB transfection, iPSCs expressed nuclear BFP and differentiated into iN cells at a rate comparable to those generated using lentiviral vectors (Figure 5B). Immunocytochemistry confirmed robust expression of TrkA, OSMR, IL31RA, TRPV1, RET, TRPA1, and PRPH by day 28 (Figure 5C). Quantification of positive cells demonstrated similar or higher efficiency when compared to lentiviral delivery, with >94% of neurons expressing pruriceptor markers (Figure 5D).

The same PB protocol was also applied to the KOLF2.1J iPSC line, yielding similar efficiency, with expression of pruriceptor markers confirmed in iPruriceptors derived from this line (Figures S5A and S5B). To further resolve cellular heterogeneity and assess transcriptional identity at single-cell resolution, we performed single-nucleus

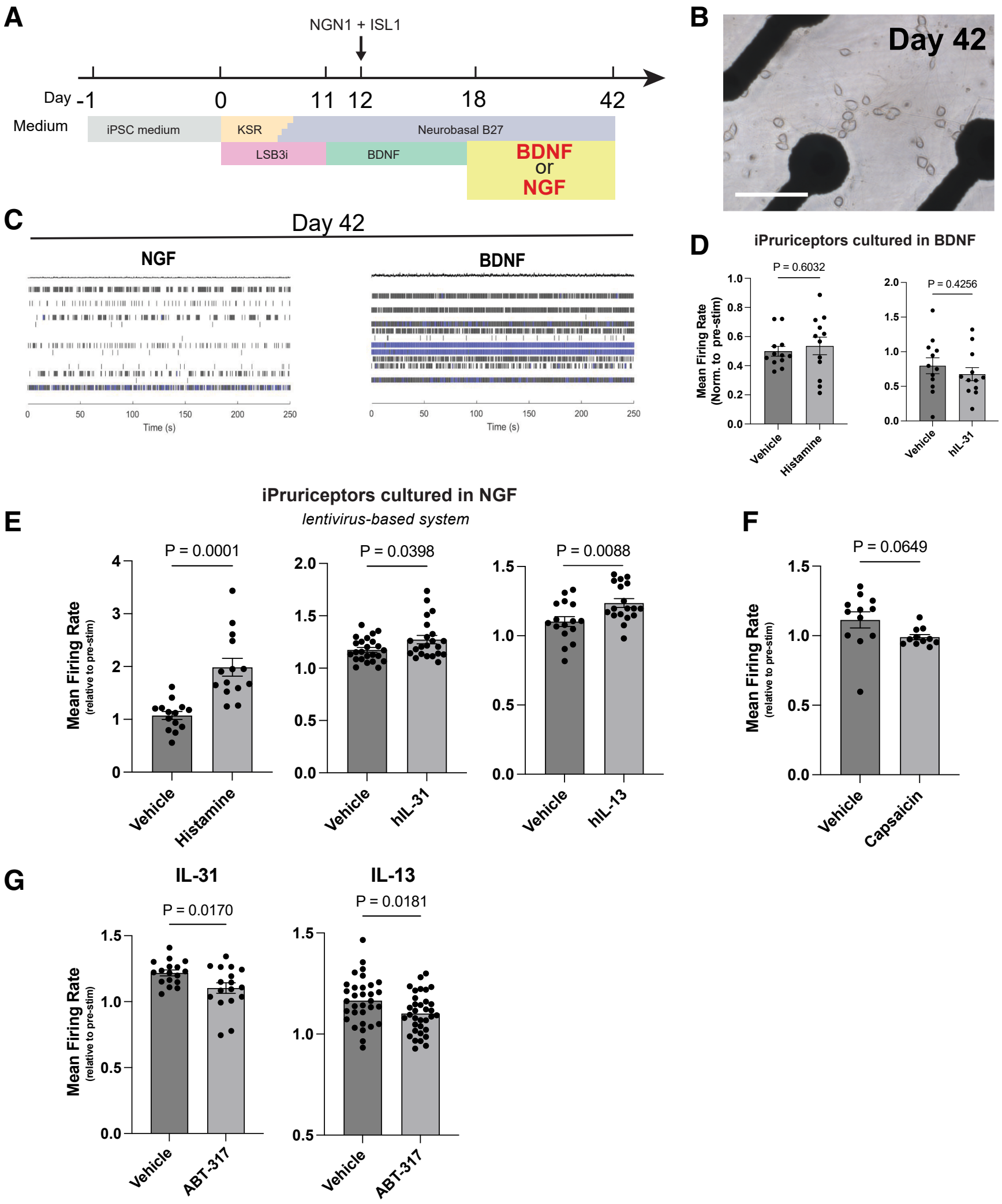

Figure 4. iPruriceptors functionally respond to pruritogenic stimuli and JAK1 inhibition

(A) Schematic showing co-expression of NGN1 and ISL1 in NCCs induced by LSB3i via lentiviral transduction, followed by culture with BDNF or NGF from day 18 to day 42, the time point of MEA analysis.

(B) Representative images showing neuronal morphology under iPruriceptor protocol on day 42 on the MEA plate. Scale bars indicate 100 μm.

(C) MEA raster plots showing neuronal spiking activity of neuronal activity on day 42 under different neurotrophic conditions (BDNF/GDNF vs. NGF) after day 18. Each vertical line represents an action potential recorded from a single electrode.

(D) MEA analysis showing neuronal responses to histamine and IL-31 (pooled from two independent experiments, $n = 6$ each) in induced neurons after day 42 from the iPruriceptor protocol maintained with BDNF/GDNF after day 18.

(E) MEA analysis showing neuronal responses to histamine (pooled from two independent experiments, $n = 6$ each), IL-31 (pooled from four independent experiments, $n = 6$ each), and IL-13 (pooled from two independent experiments, $n = 6–12$ each) after day 42 in induced neurons derived with the lentiviral-based protocol and maintained with NGF after day 18.

(F) MEA analysis showing neuronal responses to capsaicin (pooled from two independent experiments, $n = 6$ each) on day 42 in induced neurons from the iPruriceptor protocol maintained with NGF after day 18.

(G) MEA analysis showing firing rates after IL-31 (pooled from three independent experiments, $n = 6–8$ each) and IL-13 (pooled from three independent experiments, $n = 10–12$ each) after day 42 in the presence or absence of a JAK1 inhibitor, ABT-317. Values represent mean firing rates normalized to each well's pre-stimulation baseline in (D–G). Data are presented as mean ± SEM. Each dot represents one well. Stimuli were applied to independent wells (i.e., no matched or sequential stimulation within the same well), and wells were not matched across conditions. Only wells meeting predefined activity criteria were included in the analysis; therefore, the number of data points varies across conditions. Statistical comparisons between two groups were performed using an unpaired t test with Welch's correction. Also see Figure S4.

RNA sequencing (snRNA-seq) of PB-derived iPruriceptors at day 0 (iPSC stage) and day 20, prior to glial co-culture and electrophysiological maturation. Uniform manifold approximation and projection (UMAP) analysis demonstrated clear segregation of day 20 cells from the starting iPSC population (Figure 5E). Approximately 85% of day 20 cells clustered within a neuronal compartment characterized by homogeneous enrichment of pan-neuronal gene signatures. Within this neuronal population, a substantial fraction of cells exhibited enrichment for pruriceptor-associated transcriptional programs. Using an AUCell threshold of 0.15, ∼20% of day 20 cells showed enrichment for pruriceptor-associated transcription factor modules, while ∼34% and ∼28% demonstrated enrichment for human NP H11 and H10 gene signatures, respectively (Figure 5F). These findings are consistent with specification toward an NP-pruriceptor lineage. Smaller subsets of cells displayed modest enrichment for alternative sensory neuron programs, including mechanoreceptor- and proprioceptor-associated modules, indicating limited subtype

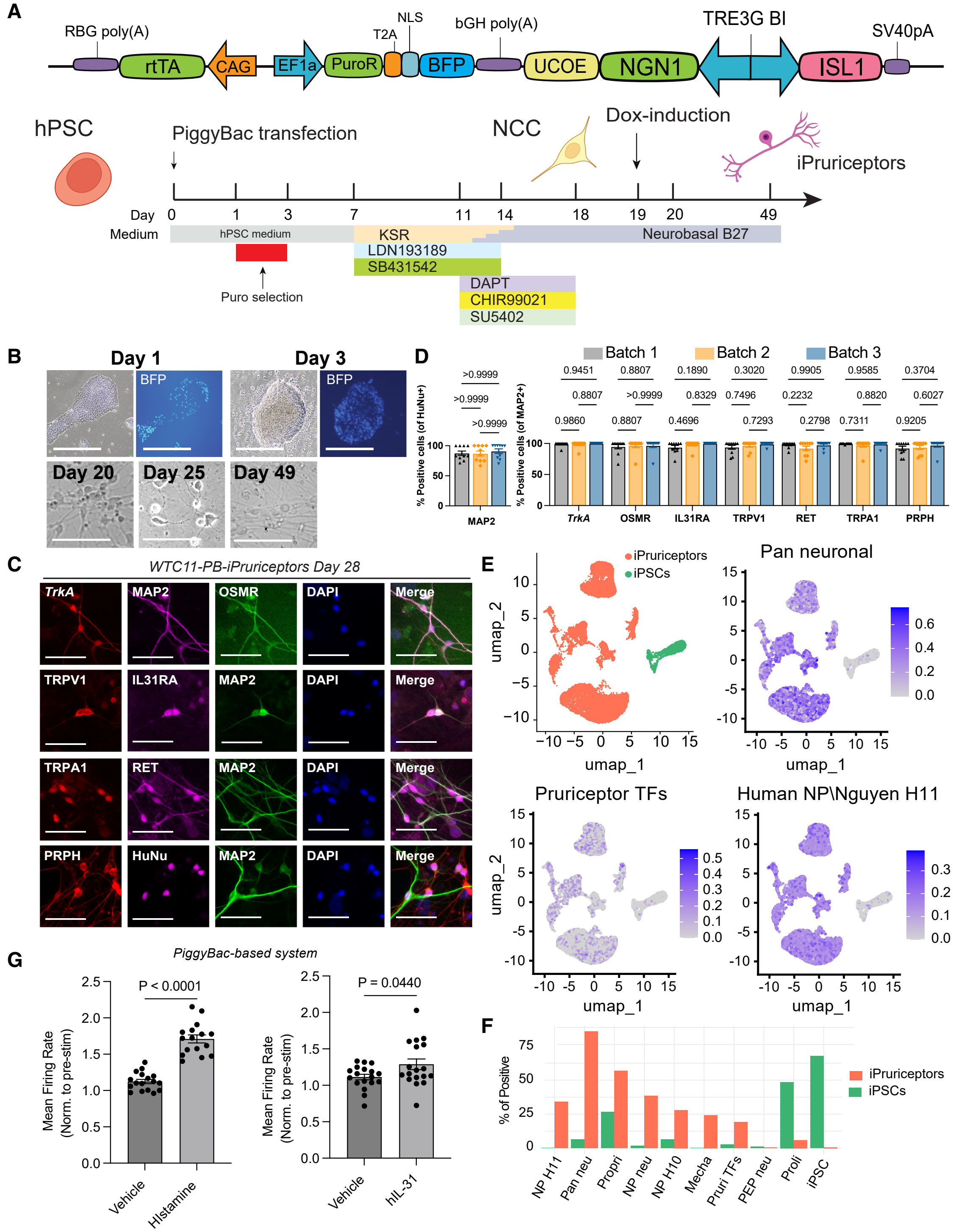

Figure 5. iPruriceptors generated with PiggyBac system reproduce robust functional pruriceptor induction

(A) Schematic of co-expression of NGN1 with ISL1 in NCCs induced by LSB3i using PiggyBac system, PB-iPruriceptor protocol.

(B) Representative images showing neuronal morphology and BFP expression at different stages of differentiation under the PB-iPruriceptor protocol on day 1, 3, 20, 25, 49. Scale bars indicate 100 μm (top) and 50 μm (bottom).

(C) Immunofluorescence staining of indicated markers on the induced neurons under iPruriceptor protocol with PiggyBac on day 28. Scale bars indicate 50 μm.

(legend continued on next page)

heterogeneity at this early developmental stage (Figure 5E; Figures S5C and S5D). Pluripotency-associated genes (*POU5F1* and *NANOG*) were nearly absent (~1%), and only a small fraction of cells (~5%) expressed proliferation-associated signatures, consistent with minimal residual undifferentiated or cycling populations. Finally, MEA recordings revealed functional responses to histamine and hIL-31 in induced neurons derived with the piggyBac-based protocol (Figure 5G). Together, these results demonstrate that functional iPruriceptors can be efficiently generated from iPSCs using a transfection-based method and without viral vectors.

DISCUSSION

To our knowledge, this is the first study reporting the induction of pruriceptors from iPSCs through the forced expression of lineage-specifying TFs. A previous study described the generation of itch sensory neuron-like cells (ISNLCs) from iPSCs using a method based solely on extrinsic factors (Guo et al., 2022). While ISNLCs express pruriceptor markers, they exhibit variability in their responses to histamine and PAR-2, lack expression of the IL-31 receptor (IL31RA), and the overall neuronal induction efficiency was not clearly quantified. Another study using extrinsic factor-based differentiation reported up to 90% efficiency in generating sensory neurons expressing IL31RA; however, functional characterization was limited to calcium influx measurements (Umehara et al., 2020).

Our protocol integrates the precision of extrinsic factors patterning with the speed and efficiency of TF-mediated reprogramming to generate iPruriceptors that recapitulate key molecular and functional features associated with human NP-pruriceptors. By inducing TFs in NCCs, rather than directly in iPSCs, our approach enhances culture synchronization and promotes neuronal maturation, achieving approximately 93% efficiency (defined as the proportion of MAP2+ and TrkA+ cells produced by the end of protocol).

The forced expression of TFs has been employed by many groups as tool to differentiate pluripotent or somatic cells into iN (Pang et al., 2011), for instance the basic-helix-loop-helix (bHLH) NGN2 has been widely used as versatile neurogenic TF—for a comprehensive review see Hulme et al. (2022)—to derive iN with features of CNS and PNS neurons including sensory neurons (Plumbly et al., 2022).

Our approach to generate iN with pruriceptor features (iPruriceptors) is based on the observation that mouse DRG sensory neuron development is initiated by a neurogenic phase induced by TFs of the neurogenin class (Fode et al., 1998; Ma et al., 1999). Both NGN1 and NGN2 and play a critical neurogenic role, as double-knockout mice fail to develop DRGs. However, these two factors have distinct roles: NGN2 is specifically required for the generation of mechanoreceptive and proprioceptive TrkC+ and TrkB+ neurons, whereas the formation of nociceptive TrkA+ neurons relies exclusively on NGN1 (Ma et al., 1999). Following the neurogenic phase, sensory neurons initiate expression of the homeodomain TFs, Brn3a and Isl1 (Eng et al., 2001; Fedtsova and Turner, 1995; Sun et al., 2008). Although Brn3a (a POU domain factor) and Islet1 (a LIM-homeodomain factor) belong to distinct TF families, they have overlapping downstream targets. Their co-expression in all sensory neurons has led to the proposal that they constitute a combinatorial code for sensory lineage specification (Anderson, 1999; Dykes et al., 2011).

Finally, Brn3a and Islt1 synergistically interact with TFs of the RUNX family to establish the gene regulatory program required for the formation of TrkC+ neurons (Orlovsky et al., 2024).

In line with these developmental roles, we found that ISL1, when co-expressed with NGN1 in NCCs, robustly induces a pruriceptor-specific transcriptional program, partly through the upregulation of *BRN3A*. However, the reverse is not true: BRN3A does not induce *ISL1* expression nor does it efficiently drive sensory neuron fate on its own. This finding is surprising, particularly because BRN3A has previously been used in combination with NGN2 to generate mixed populations of DRG-like sensory neurons

(D) Bar graph showing the proportion of induced neurons under iPruriceptor protocol with PiggyBac expressing the indicated markers on day 28. Data are presented as mean ± SEM across three independent batches. Each dot represents one field of view. Statistical comparisons between the batches were performed using a two-way ANOVA test.
(E) UMAP visualization showing separation between iPSCs and iPruriceptors in snRNA-seq datasets. Cell identities were inferred using UCell gene-program scoring for neuronal, pruriceptor signatures described in Figure S5D.
(F) Quantification of the proportion of positive cells for each signature (UCell score >0.15) in iPSCs and iPruriceptors cultures, highlighting enrichment of neuronal, pruriceptor-related signatures in iPruriceptors.
(G) MEA analysis showing histamine- and IL-31-induced neuronal responses on day 49 in induced neurons derived with the PiggyBac-based protocol and maintained with NGF after day 18. The values represent the mean firing rate, normalized to the baseline firing rate before stimulation for each well. Data are presented as mean ± SEM. Each dot represents one well. Data are pooled from three independent experiments, n = 4–12 each). Statistical comparisons between the two groups were performed using an unpaired t test with Welch's correction. Also see Figure S5.

from fibroblasts, iPSCs, and NCCs (Blanchard et al., 2015; Nickolls et al., 2020). A likely explanation lies in the context-dependent functions of BRN3A, which may act in concert with neuron subtype-specific cofactors. In fact, BRN3A is known to function as a terminal selector of neuronal identity in the CNS (Serrano-Saiz et al., 2018), and a hierarchical relationship between ISL1 and BRN3A has also been observed during inner ear neuron development, where ISL1 expression precedes and possibly regulates BRN3A (Deng et al., 2014; Xu et al., 2024).

Similarly, the observation that RUNX1 is inefficient at inducing iPruriceptors is unexpected but can be explained by its position downstream of ISL1 and BRN3A in the transcriptional hierarchy. RUNX1 is known to refine nociceptor subtype identity rather than initiate sensory lineage specification. When co-expressed with an early neurogenic factor like NGN1, RUNX1 may fail to activate an appropriate pruriceptor gene expression program. This is consistent with its known developmental dynamics: RUNX1 is initially activated by ISL1 and BRN3A but later represses early sensory markers such as TrkA to promote subtype diversification (Chen et al., 2006). RUNX1's dual role—acting first as a transcriptional activator and later as a repressor—adds complexity that may limit its utility in simplified *in vitro* differentiation models such as ours.

One key finding of this study is that pruriceptor differentiation is critically dependent on the NGF signaling environment. While most NGN2-iN models supplement their media with BDNF and Neurotrophin-3 (NT3) (Bullmann et al., 2024; Zhang et al., 2013), we observed that iPruriceptors only became functionally responsive to pruritogens when cultured in NGF-containing media, rather than BDNF. This aligns with *in vivo* studies demonstrating that NGF promotes nociceptive lineage maturation (Lu et al., 2024), while BDNF primarily supports mechanoreceptors and proprioceptors. The requirement for NGF supplementation highlights the complex interplay between extrinsic neurotrophic signals and intrinsic transcriptional programs in sensory neuron differentiation. snRNA-seq analysis confirmed enrichment for NP H10/H11 pruriceptor signatures and canonical pruriceptor-associated genes, with minimal residual pluripotent or proliferative populations. While the cultures are enriched for NP-pruriceptor transcriptional programs, modest enrichment of alternative sensory neuron modules likely reflects developmental immaturity and shared early transcriptional programs among DRG subtypes rather than substantial contamination by unrelated sensory lineages. A major strength of our study is the functional validation of iPruriceptors in a pharmacologically relevant context. Using MEA recordings, we demonstrated that iPruriceptors exhibit pruritogen-induced activity, which can be pharmacologically suppressed by the JAK1-selective inhibitor ABT-317. This finding has direct clinical relevance, as JAK1-selective inhibition is now a proven therapeutic strategy for chronic pruritus in multiple conditions (Kwatra et al., 2024; Simpson et al., 2024). The ability of our model to recapitulate drug responses observed in patients positions it as a promising platform for drug discovery and mechanistic studies of itch-related disorders. Of note, robust electrophysiological maturation required astrocyte co-culture, consistent with other transcription factor-driven neuronal systems (Yang et al., 2017; Zhang et al., 2013). Mouse primary astrocytes were used to provide standardized trophic support and enable species-specific transcript separation during RNA-seq analyses. Furthermore, the PB transposon-based expression system offers an efficient and scalable method for generating iPruriceptors, bypassing the limitations associated with viral transduction. This innovation enhances the translational potential of our protocol, facilitating high-throughput screening of pruritic and anti-pruritic compounds.

While the current results show that our protocol reliably generates iPruriceptors, its validation remains limited to two iPSC lines, as such, its robustness across a broader range of iPSC lines has yet to be established. Single-nucleus transcriptomic analysis was performed at day 20 to assess early transcriptional specification prior to glial co-culture and electrophysiological maturation. While this provides resolution of lineage commitment and heterogeneity at this stage, *in vitro*-derived sensory neurons likely continue to refine subtype identity over time, and complete equivalence to adult human pruriceptors cannot be assumed. Also, while ISL1 co-expression improved pruriceptor specification, additional TFs, such as ETV1, ELF1, FOXO3, FOXN3, and KLF5 may further refine differentiation. Future studies should explore whether sequential expression of NGN1/ISL1 followed by these TFs could enhance pruriceptor purity and functional maturation. Another key consideration is the absence of a fully reconstructed itch circuit *in vitro*. Although our model enables the study of peripheral neuron responses, future work should integrate co-culture systems with keratinocytes, immune cells, and spinal cord neurons to better mimic *in vivo* pruriceptive pathways. More studies will be necessary to further optimize and adapt this protocol for true high-throughput applications and to develop a robust drug screening platform suitable for preclinical interrogation of compounds targeting pruriceptor activity. A key goal will be the generation of iPruriceptors from a diverse panel of iPSC lines derived from both healthy individuals and patients with atopic dermatitis (AD) and CPUO. This will enable the identification of functional, disease-specific phenotypes and allow for personalized therapeutic targeting.

RESOURCE AVAILABILITY

Lead contact

Further information and requests for resources and reagents should be directed to the lead contact, Samuele G. Marro (samuele.marro@mssm.edu).

Materials availability

All unique reagents generated in this study are available from the lead contact upon reasonable request and completion of a materials transfer agreement. Plasmids generated in this study have been deposited at Addgene.

Data and code availability

- Data reported in this paper will be shared by the lead contact upon request.
- This paper does not report original code.
- RNA sequencing data have been deposited in the Gene Expression Omnibus (GEO) repository with accession number GSE329269.

ACKNOWLEDGMENTS

This work was supported by the NIH-NINDS R21NS130319 and Cindy Silvian Foundation Grant to S.G.M. Experiments were supported by the Stem Cell Engineering Core (RRID: SCR_027503) at the Icahn School of Medicine at Mount Sinai. The authors also thank Jenna Chan for her assistance with cloning.

AUTHOR CONTRIBUTIONS

Conceptualization, S.G.M., B.S.K., E.R.G., P.R., H.I., and R.H.; data acquisition, H.I., R.H., X.L., E.B., C.H., I.I., L.N.M., K.C., and D.Y.; RNA-seq library preparation, R.K.; data analysis and interpretation, A.V.H. and K.M.S.; manuscript draft, S.G.M. and H.I.; manuscript review and editing, all authors.

DECLARATION OF INTERESTS

The authors declare no competing interests.

STAR★METHODS

Detailed methods are provided in the online version of this paper and include the following:

- KEY RESOURCES TABLE
- METHOD DETAILS
  - iPSCs maintenance
  - Cell line quality control
  - Plasmids
  - Lentivirus packaging
  - Generation of iPSC lines with PiggyBac-integrated transgenes
  - Neural induction using forced expression of TFs with lentivirus on iPSC (one-step)
  - Neural induction using forced expression of TFs with lentivirus or PiggyBac on NCCs (2-step)
  - RNA extraction and quantitative real-time PCR
  - Bulk RNA-seq
  - Single-nucleus RNA sequencing (snRNA-seq)
  - Multielectrode array (MEA)
  - Immunofluorescence staining
  - Mice
  - Mouse glia cell preparation

SUPPLEMENTAL INFORMATION

Supplemental information can be found online at https://doi.org/10.1016/j.stemcr.2026.103004.

Received: September 29, 2025
Revised: June 1, 2026
Accepted: June 8, 2026
Published: July 9, 2026

REFERENCES

Anderson, D.J. (1999). Lineages and transcription factors in the specification of vertebrate primary sensory neurons. Curr. Opin. Neurobiol. *9*, 517–524. https://doi.org/10.1016/S0959-4388(99)00015-X.

Andreatta, M., and Carmona, S.J. (2021). UCell: Robust and scalable single-cell gene signature scoring. Comput. Struct. Biotechnol. J. *19*, 3796–3798. https://doi.org/10.1016/j.csbj.2021.06.043.

Bankhead, P., Loughrey, M.B., Fernández, J.A., Dombrowski, Y., McArt, D.G., Dunne, P.D., McQuaid, S., Gray, R.T., Murray, L.J., Coleman, H.G., et al. (2017). QuPath: Open source software for digital pathology image analysis. Sci. Rep. *7*, 16878. https://doi.org/10.1038/s41598-017-17204-5.

Blanchard, J.W., Eade, K.T., Szűcs, A., Lo Sardo, V., Tsunemoto, R.K., Williams, D., Sanna, P.P., and Baldwin, K.K. (2015). Selective conversion of fibroblasts into peripheral sensory neurons. Nat. Neurosci. *18*, 25–35. https://doi.org/10.1038/nn.3887.

Bray, N.L., Pimentel, H., Melsted, P., and Pachter, L. (2016). Near-optimal probabilistic RNA-seq quantification. Nat. Biotechnol. *34*, 525–527. https://doi.org/10.1038/nbt.3519.

Bullmann, T., Kaas, T., Ritzau-Jost, A., Wöhner, A., Kirmann, T., Rizalar, F.S., Holzer, M., Nerlich, J., Puchkov, D., Geis, C., et al. (2024). Human iPSC-Derived Neurons with Reliable Synapses and Large Presynaptic Action Potentials. J. Neurosci. *44*, e0971232024. https://doi.org/10.1523/JNEUROSCI.0971-23.2024.

Butler, D.C., Berger, T., Elmariah, S., Kim, B., Chisolm, S., Kwatra, S.G., Mollanazar, N., and Yosipovitch, G. (2024). Chronic Pruritus: A Review. JAMA *331*, 2114–2124. https://doi.org/10.1001/jama.2024.4899.

Chambers, S.M., Fasano, C.A., Papapetrou, E.P., Tomishima, M., Sadelain, M., and Studer, L. (2009). Highly efficient neural conversion of human ES and iPS cells by dual inhibition of SMAD signaling. Nat. Biotechnol. *27*, 275–280. https://doi.org/10.1038/nbt.1529.

Chambers, S.M., Qi, Y., Mica, Y., Lee, G., Zhang, X.J., Niu, L., Bilsland, J., Cao, L., Stevens, E., Whiting, P., et al. (2012). Combined small-molecule inhibition accelerates developmental timing and

converts human pluripotent stem cells into nociceptors. Nat. Biotechnol. *30*, 715–720. https://doi.org/10.1038/nbt.2249.

Chen, C.L., Broom, D.C., Liu, Y., de Nooij, J.C., Li, Z., Cen, C., Samad, O.A., Jessell, T.M., Woolf, C.J., and Ma, Q. (2006). Runx1 determines nociceptive sensory neuron phenotype and is required for thermal and neuropathic pain. Neuron *49*, 365–377. https://doi.org/10.1016/j.neuron.2005.10.036.

Chen, M., Maimaitili, M., Habekost, M., Gill, K.P., Mermet-Joret, N., Nabavi, S., Febbraro, F., and Denham, M. (2020). Rapid generation of regionally specified CNS neurons by sequential patterning and conversion of human induced pluripotent stem cells. Stem Cell Res. *48*, 101945. https://doi.org/10.1016/j.scr.2020.101945.

Defaye, M., Iftinca, M.C., Gadotti, V.M., Basso, L., Abdullah, N.S., Cuménal, M., Agosti, F., Hassan, A., Flynn, R., Martin, J., et al. (2022). The neuronal tyrosine kinase receptor ligand ALKAL2 mediates persistent pain. J. Clin. Investig. *132*, e154317. https://doi.org/10.1172/JCI154317.

Deng, M., Yang, H., Xie, X., Liang, G., and Gan, L. (2014). Comparative expression analysis of POU4F1, POU4F2 and ISL1 in developing mouse cochleovestibular ganglion neurons. Gene Expr. Patterns *15*, 31–37. https://doi.org/10.1016/j.gep.2014.03.001.

Dobin, A., Davis, C.A., Schlesinger, F., Drenkow, J., Zaleski, C., Jha, S., Batut, P., Chaisson, M., and Gingeras, T.R. (2013). STAR: ultrafast universal RNA-seq aligner. Bioinformatics *29*, 15–21. https://doi.org/10.1093/bioinformatics/bts635.

Dull, T., Zufferey, R., Kelly, M., Mandel, R.J., Nguyen, M., Trono, D., and Naldini, L. (1998). A third-generation lentivirus vector with a conditional packaging system. J. Virol. *72*, 8463–8471. https://doi.org/10.1128/JVI.72.11.8463-8471.1998.

Durens, M., Baljinnyam, E., Grisanti, L., Hu, R., and Marro, S.G. (2024). An induced pluripotent stem cell line carrying a silencing-resistant calcium reporter allele. Stem Cell Res. *79*, 103455. https://doi.org/10.1016/j.scr.2024.103455.

Dykes, I.M., Tempest, L., Lee, S.I., and Turner, E.E. (2011). Brn3a and Islet1 act epistatically to regulate the gene expression program of sensory differentiation. J. Neurosci. *31*, 9789–9799. https://doi.org/10.1523/JNEUROSCI.0901-11.2011.

Eng, S.R., Gratwick, K., Rhee, J.M., Fedtsova, N., Gan, L., and Turner, E.E. (2001). Defects in sensory axon growth precede neuronal death in Brn3a-deficient mice. J. Neurosci. *21*, 541–549. https://doi.org/10.1523/JNEUROSCI.21-02-00541.2001.

Fedtsova, N.G., and Turner, E.E. (1995). Brn-3.0 expression identifies early post-mitotic CNS neurons and sensory neural precursors. Mech. Dev. *53*, 291–304. https://doi.org/10.1016/0925-4773(95)00435-1.

Fode, C., Gradwohl, G., Morin, X., Dierich, A., LeMeur, M., Goridis, C., and Guillemot, F. (1998). The bHLH protein NEUROGENIN 2 is a determination factor for epibranchial placode-derived sensory neurons. Neuron *20*, 483–494. https://doi.org/10.1016/s0896-6273(00)80989-7.

Ge, T., Jhala, G., Fynch, S., Akazawa, S., Litwak, S., Pappas, E.G., Catterall, T., Vakil, I., Long, A.J., Olson, L.M., et al. (2020). The JAK1 Selective Inhibitor ABT 317 Blocks Signaling Through Interferon-gamma and Common gamma Chain Cytokine Receptors to Reverse Autoimmune Diabetes in NOD Mice. Front. Immunol. *11*, 588543. https://doi.org/10.3389/fimmu.2020.588543.

Goparaju, S.K., Kohda, K., Ibata, K., Soma, A., Nakatake, Y., Akiyama, T., Wakabayashi, S., Matsushita, M., Sakota, M., Kimura, H., et al. (2017). Rapid differentiation of human pluripotent stem cells into functional neurons by mRNAs encoding transcription factors. Sci. Rep. *7*, 42367. https://doi.org/10.1038/srep42367.

Gu, J., Sumer, H., and Cromer, B. (2022). Efficient Generation of Stable Cell Lines with Inducible Neuronal Transgene Expression Using the piggyBac Transposon System. Methods Mol. Biol. *2495*, 49–66. https://doi.org/10.1007/978-1-0716-2301-5_3.

Guo, Z., Tong, C.K., Jacków, J., Doucet, Y.S., Abaci, H.E., Zeng, W., Hansen, C., Hayashi, R., DeLorenzo, D., Rami, A., et al. (2022). Engineering human skin model innervated with itch sensory neuron-like cells differentiated from induced pluripotent stem cells. Bioeng. Transl. Med. *7*, e10247. https://doi.org/10.1002/btm2.10247.

Hao, Y., Hao, S., Andersen-Nissen, E., Mauck, W.M., 3rd, Zheng, S., Butler, A., Lee, M.J., Wilk, A.J., Darby, C., Zager, M., et al. (2021). Integrated analysis of multimodal single-cell data. Cell *184*, 3573–3587.e29. https://doi.org/10.1016/j.cell.2021.04.048.

Holzer, A.K., Karreman, C., Suciu, I., Furmanowsky, L.S., Wohlfarth, H., Loser, D., Dirks, W.G., Pardo González, E., and Leist, M. (2022). Generation of Human Nociceptor-Enriched Sensory Neurons for the Study of Pain-Related Dysfunctions. Stem Cells Transl. Med. *11*, 727–741. https://doi.org/10.1093/stcltm/szac031.

Huang, D., Lu, J., and Tan, F. (2024). Improvement of Pruritus and Efficacy in the Early Stage of Therapy with Upadacitinib, Abrocitinib, or Dupilumab in Chinese Patients with Moderate-to-Severe Atopic Dermatitis: Prospective, Cohort, Observational Study. Dermatitis *35*, 77–83. https://doi.org/10.1089/derm.2023.0132.

Hulme, A.J., Maksour, S., St-Clair Glover, M., Miellet, S., and Dottori, M. (2022). Making neurons, made easy: The use of Neurogenin-2 in neuronal differentiation. Stem Cell Rep. *17*, 14–34. https://doi.org/10.1016/j.stemcr.2021.11.015.

Jeon, J., Wang, F., Badic, A., and Kim, B.S. (2022). Treatment of patients with chronic pruritus of unknown origin with dupilumab. J. Dermatol. Treat. *33*, 1754–1757. https://doi.org/10.1080/09546634.2021.1880542.

Jung, M., Dourado, M., Maksymetz, J., Jacobson, A., Laufer, B.I., Baca, M., Foreman, O., Hackos, D.H., Riol-Blanco, L., and Kaminker, J.S. (2023). Cross-species transcriptomic atlas of dorsal root ganglia reveals species-specific programs for sensory function. Nat. Commun. *14*, 366. https://doi.org/10.1038/s41467-023-36014-0.

Kim, B.S., Berger, T.G., and Yosipovitch, G. (2019). Chronic pruritus of unknown origin (CPUO): Uniform nomenclature and diagnosis as a pathway to standardized understanding and treatment. J. Am. Acad. Dermatol. *81*, 1223–1224. https://doi.org/10.1016/j.jaad.2019.06.038.

Kim, B.S., Silverberg, J.I., Ständer, S., Yosipovitch, G., Simpson, E.L., DiBonaventura, M., Kerkmann, U., Farooqui, S.A., Biswas, P., Valdez, H., and Cameron, M.C. (2021). Rapid Improvement of Itch Associated With Atopic Dermatitis With Abrocitinib Is Partially Independent of Overall Disease Improvement: Results

From Pooled Phase 2b and 3 Monotherapy Studies. Dermatitis *32*, S39–S44. https://doi.org/10.1097/DER.0000000000000770.

Kim, B.S., Sun, K., Papp, K., Venturanza, M., Nasir, A., and Kuligowski, M.E. (2020). Effects of ruxolitinib cream on pruritus and quality of life in atopic dermatitis: Results from a phase 2, randomized, dose-ranging, vehicle- and active-controlled study. J. Am. Acad. Dermatol. *82*, 1305–1313. https://doi.org/10.1016/j.jaad.2020.02.009.

Kwatra, S.G., Bordeaux, Z.A., Parthasarathy, V., Kollhoff, A.L., Alajmi, A., Pritchard, T., Cornman, H.L., Kambala, A., Lee, K.K., Manjunath, J., et al. (2024). Efficacy and Safety of Abrocitinib in Prurigo Nodularis and Chronic Pruritus of Unknown Origin: A Nonrandomized Controlled Trial. JAMA Dermatol. *160*, 717–724. https://doi.org/10.1001/jamadermatol.2024.1464.

Lallemend, F., and Ernfors, P. (2012). Molecular interactions underlying the specification of sensory neurons. Trends Neurosci. *35*, 373–381. https://doi.org/10.1016/j.tins.2012.03.006.

Liput, D.J., Lu, V.B., Davis, M.I., Puhl, H.L., and Ikeda, S.R. (2016). Rem2, a member of the RGK family of small GTPases, is enriched in nuclei of the basal ganglia. Sci. Rep. *6*, 25137. https://doi.org/10.1038/srep25137.

Love, M.I., Huber, W., and Anders, S. (2014). Moderated estimation of fold change and dispersion for RNA-seq data with DESeq2. Genome Biol. *15*, 550. https://doi.org/10.1186/s13059-014-0550-8.

Lu, T., Wang, M., Zhou, W., Ni, Q., Yue, Y., Wang, W., Shi, Y., Liu, Z., Li, C., Hong, B., et al. (2024). Decoding transcriptional identity in developing human sensory neurons and organoid modeling. Cell *187*, 7374–7393.e28. https://doi.org/10.1016/j.cell.2024.10.023.

Ma, Q., Fode, C., Guillemot, F., and Anderson, D.J. (1999). Neurogenin1 and neurogenin2 control two distinct waves of neurogenesis in developing dorsal root ganglia. Genes Dev. *13*, 1717–1728. https://doi.org/10.1101/gad.13.13.1717.

Marmigère, F., and Ernfors, P. (2007). Specification and connectivity of neuronal subtypes in the sensory lineage. Nat. Rev. Neurosci. *8*, 114–127. https://doi.org/10.1038/nrn2057.

Marro, S.G., Chanda, S., Yang, N., Janas, J.A., Valperga, G., Trotter, J., Zhou, B., Merrill, S., Yousif, I., Shelby, H., et al. (2019). Neuroligin-4 Regulates Excitatory Synaptic Transmission in Human Neurons. Neuron *103*, 617–626.e6. https://doi.org/10.1016/j.neuron.2019.05.043.

Meng, Q.T., Liu, X.Y., Liu, X.T., Liu, J., Munanairi, A., Barry, D.M., Liu, B., Jin, H., Sun, Y., Yang, Q., et al. (2021). BNP facilitates NMB-encoded histaminergic itch via NPRC-NMBR crosstalk. eLife *10*, e71689. https://doi.org/10.7554/eLife.71689.

Mica, Y., Lee, G., Chambers, S.M., Tomishima, M.J., and Studer, L. (2013). Modeling neural crest induction, melanocyte specification, and disease-related pigmentation defects in hESCs and patient-specific iPSCs. Cell Rep. *3*, 1140–1152. https://doi.org/10.1016/j.celrep.2013.03.025.

Mishra, S.K., and Hoon, M.A. (2013). The cells and circuitry for itch responses in mice. Science *340*, 968–971. https://doi.org/10.1126/science.1233765.

Müller-Kuller, U., Ackermann, M., Kolodziej, S., Brendel, C., Fritsch, J., Lachmann, N., Kunkel, H., Lausen, J., Schambach, A., Moritz, T., and Grez, M. (2015). A minimal ubiquitous chromatin opening element (UCOE) effectively prevents silencing of juxtaposed heterologous promoters by epigenetic remodeling in multipotent and pluripotent stem cells. Nucleic Acids Res. *43*, 1577–1592. https://doi.org/10.1093/nar/gkv019.

Nguyen, M.Q., von Buchholtz, L.J., Reker, A.N., Ryba, N.J., and Davidson, S. (2021). Single-nucleus transcriptomic analysis of human dorsal root ganglion neurons. eLife *10*, e71752. https://doi.org/10.7554/eLife.71752.

Nickolls, A.R., Lee, M.M., Espinoza, D.F., Szczot, M., Lam, R.M., Wang, Q., Beers, J., Zou, J., Nguyen, M.Q., Solinski, H.J., et al. (2020). Transcriptional Programming of Human Mechanosensory Neuron Subtypes from Pluripotent Stem Cells. Cell Rep. *30*, 932–946.e7. https://doi.org/10.1016/j.celrep.2019.12.062.

Oetjen, L.K., Mack, M.R., Feng, J., Whelan, T.M., Niu, H., Guo, C.J., Chen, S., Trier, A.M., Xu, A.Z., Tripathi, S.V., et al. (2017). Sensory Neurons Co-opt Classical Immune Signaling Pathways to Mediate Chronic Itch. Cell *171*, 217–228.e13. https://doi.org/10.1016/j.cell.2017.08.006.

Orlovsky, K., Appel, E., Hantisteanu, S., Olender, T., Lotem, J., Levanon, D., and Groner, Y. (2024). Runx3, Brn3a and Isl1 interplay orchestrates the transcriptional program in the early stages of proprioceptive neuron development. PLoS Genet. *20*, e1011401. https://doi.org/10.1371/journal.pgen.1011401.

Pang, Z.P., Yang, N., Vierbuchen, T., Ostermeier, A., Fuentes, D.R., Yang, T.Q., Citri, A., Sebastiano, V., Marro, S., Südhof, T.C., and Wernig, M. (2011). Induction of human neuronal cells by defined transcription factors. Nature *476*, 220–223. https://doi.org/10.1038/nature10202.

Pantazis, C.B., Yang, A., Lara, E., McDonough, J.A., Blauwendraat, C., Peng, L., Oguro, H., Kanaujiya, J., Zou, J., Sebesta, D., et al. (2022). A reference human induced pluripotent stem cell line for large-scale collaborative studies. Cell Stem Cell *29*, 1685–1702.e1622. https://doi.org/10.1016/j.stem.2022.11.004.

Plumbly, W., Patikas, N., Field, S.F., Foskolou, S., and Metzakopian, E. (2022). Derivation of nociceptive sensory neurons from hiPSCs with early patterning and temporally controlled NEUROG2 overexpression. Cell Rep Methods *2*, 100341. https://doi.org/10.1016/j.crmeth.2022.100341.

Rosa, F.F., Pires, C.F., Kurochkin, I., Ferreira, A.G., Gomes, A.M., Palma, L.G., Shaiv, K., Solanas, L., Azenha, C., Papatsenko, D., et al. (2018). Direct reprogramming of fibroblasts into antigen-presenting dendritic cells. Sci. Immunol. *3*, eaau4292. https://doi.org/10.1126/sciimmunol.aau4292.

Satija, R., Farrell, J.A., Gennert, D., Schier, A.F., and Regev, A. (2015). Spatial reconstruction of single-cell gene expression data. Nat. Biotechnol. *33*, 495–502. https://doi.org/10.1038/nbt.3192.

Schrenk-Siemens, K., Pohle, J., Rostock, C., Abd El Hay, M., Lam, R.M., Szczot, M., Lu, S., Chesler, A.T., and Siemens, J. (2022). Human Stem Cell-Derived TRPV1-Positive Sensory Neurons: A New Tool to Study Mechanisms of Sensitization. Cells *11*, 2905. https://doi.org/10.3390/cells11182905.

Serrano-Saiz, E., Leyva-Díaz, E., De La Cruz, E., and Hobert, O. (2018). BRN3-type POU Homeobox Genes Maintain the Identity of Mature Postmitotic Neurons in Nematodes and Mice. Curr.

Biol. *28*, 2813–2823.e2. https://doi.org/10.1016/j.cub.2018.06.045.

Simpson, E.L., Prajapati, V.H., Leshem, Y.A., Chovatiya, R., de Bruin-Weller, M.S., Ständer, S., Pink, A.E., Calimlim, B.M., Lee, W.J., Teixeira, H., et al. (2024). Upadacitinib Rapidly Improves Patient-Reported Outcomes in Atopic Dermatitis: 16-Week Results from Phase 3 Clinical Trials (Measure Up 1 and 2). Dermatol. Ther. *14*, 1127–1144. https://doi.org/10.1007/s13555-024-01157-5.

Sommer, L., Ma, Q., and Anderson, D.J. (1996). neurogenins, a novel family of atonal-related bHLH transcription factors, are putative mammalian neuronal determination genes that reveal progenitor cell heterogeneity in the developing CNS and PNS. Mol. Cell. Neurosci. *8*, 221–241. https://doi.org/10.1006/mcne.1996.0060.

Stewart, S.A., Dykxhoorn, D.M., Palliser, D., Mizuno, H., Yu, E.Y., An, D.S., Sabatini, D.M., CHEN, I.S.y., Hahn, W.C., Sharp, P.A., et al. (2003). Lentivirus-delivered stable gene silencing by RNAi in primary cells. RNA *9*, 493–501. https://doi.org/10.1261/rna.2192803.

Sun, Y., Dykes, I.M., Liang, X., Eng, S.R., Evans, S.M., and Turner, E.E. (2008). A central role for Islet1 in sensory neuron development linking sensory and spinal gene regulatory programs. Nat. Neurosci. *11*, 1283–1293. https://doi.org/10.1038/nn.2209.

Tirosh, I., Venteicher, A.S., Hebert, C., Escalante, L.E., Patel, A.P., Yizhak, K., Fisher, J.M., Rodman, C., Mount, C., Filbin, M.G., et al. (2016). Single-cell RNA-seq supports a developmental hierarchy in human oligodendroglioma. Nature *539*, 309–313. https://doi.org/10.1038/nature20123.

Todorović, V., McDonald, H.A., Hoover, P., Wetter, J.B., Marinopoulos, A.E., Woody, C.L., Miller, L., Finkielsztein, A., Dunstan, R.W., Paller, A.S., et al. (2022). Cytokine induced 3-D organotypic psoriasis skin model demonstrates distinct roles for NF-kappaB and JAK pathways in disease pathophysiology. Exp. Dermatol. *31*, 1036–1047. https://doi.org/10.1111/exd.14551.

Twomey, R.E., Perper, S.J., Westmoreland, S.V., Srinivasan, S., Mathieu, S.L., Frank, K.E., Karman, J., Long, A.J., Housley, W.J., and Clarke, S.H. (2024). Therapeutic JAK1 Inhibition Reverses Lupus Nephritis in a Mouse Model and Demonstrates Transcriptional Changes Consistent With Human Disease. ACR Open Rheumatol. *6*, 900–911. https://doi.org/10.1002/acr2.11745.

Umehara, Y., Toyama, S., Tominaga, M., Matsuda, H., Takahashi, N., Kamata, Y., Niyonsaba, F., Ogawa, H., and Takamori, K. (2020). Robust induction of neural crest cells to derive peripheral sensory neurons from human induced pluripotent stem cells. Sci. Rep. *10*, 4360. https://doi.org/10.1038/s41598-020-60036-z.

Urlinger, S., Baron, U., Thellmann, M., Hasan, M.T., Bujard, H., and Hillen, W. (2000). Exploring the sequence space for tetracycline-dependent transcriptional activators: novel mutations yield expanded range and sensitivity. Proc. Natl. Acad. Sci. USA *97*, 7963–7968. https://doi.org/10.1073/pnas.130192197.

Usoskin, D., Furlan, A., Islam, S., Abdo, H., Lönnerberg, P., Lou, D., Hjerling-Leffler, J., Haeggström, J., Kharchenko, O., Kharchenko, P.V., et al. (2015). Unbiased classification of sensory neuron types by large-scale single-cell RNA sequencing. Nat. Neurosci. *18*, 145–153. https://doi.org/10.1038/nn.3881.

Wang, F., Trier, A.M., Li, F., Kim, S., Chen, Z., Chai, J.N., Mack, M.R., Morrison, S.A., Hamilton, J.D., Baek, J., et al. (2021). A basophil-neuronal axis promotes itch. Cell *184*, 422–440.e17. https://doi.org/10.1016/j.cell.2020.12.033.

Xu, M., Li, S., Xie, X., Guo, L., Yu, D., Zhuo, J., Lin, J., Kol, L., and Gan, L. (2024). ISL1 and POU4F1 Directly Interact to Regulate the Differentiation and Survival of Inner Ear Sensory Neurons. J. Neurosci. *44*, e1718232024. https://doi.org/10.1523/JNEUROSCI.1718-23.2024.

Yang, N., Chanda, S., Marro, S., Ng, Y.H., Janas, J.A., Haag, D., Ang, C.E., Tang, Y., Flores, Q., Mall, M., et al. (2017). Generation of pure GABAergic neurons by transcription factor programming. Nat. Methods *14*, 621–628. https://doi.org/10.1038/nmeth.4291.

Yosipovitch, G., Rosen, J.D., and Hashimoto, T. (2018). Itch: From mechanism to (novel) therapeutic approaches. J. Allergy Clin. Immunol. *142*, 1375–1390. https://doi.org/10.1016/j.jaci.2018.09.005.

Zhang, Y., Pak, C., Han, Y., Ahlenius, H., Zhang, Z., Chanda, S., Marro, S., Patzke, C., Acuna, C., Covy, J., et al. (2013). Rapid single-step induction of functional neurons from human pluripotent stem cells. Neuron *78*, 785–798. https://doi.org/10.1016/j.neuron.2013.05.029.

Zirlinger, M., Lo, L., McMahon, J., McMahon, A.P., and Anderson, D.J. (2002). Transient expression of the bHLH factor neurogenin-2 marks a subpopulation of neural crest cells biased for a sensory but not a neuronal fate. Proc. Natl. Acad. Sci. USA *99*, 8084–8089. https://doi.org/10.1073/pnas.122231199.

STAR★METHODS

KEY RESOURCES TABLE

REAGENT or RESOURCE	SOURCE	IDENTIFIER
**Chemicals, peptides, and recombinant proteins**		
Geltrex Reduced-Growth Factor Basement-Membrane Matrix, LDEV-free	Thermo Fisher Scientific	A1413302
StemFlex Medium	Thermo Fisher Scientific	A3349401
Chroman1	Bio-techne	7163
Accutase	Innovative Cell Technologies	AT104
Bambanker	Fujifilm Irvine Scientific	302-14686
Dulbecco's phosphate-buffered saline (DPBS)	Thermo Fisher Scientific	14190094
EDTA stock solution (0.5M)	Thermo Fisher Scientific	BP2482-500
DMEM, high glucose, HEPES	Thermo Fisher Scientific	12430062
DMEM/F-12	Thermo Fisher Scientific	11320033
Calf Serum	Fisher Scientific	SH30087.04
Sodium pyruvate	Thermo Fisher Scientific	11360070
MEM Non-Essential Amino Acids (NEAA)	Thermo Fisher Scientific	11140050
2-Mercaptoethanol 14.3M	Millipore Sigma	M3148
Poly-L-ornithine hydrobromide	Millipore Sigma	P3655
Polyethylenimine (PEI 25K)	Polysciences	23966
Lipofectamine Stem Transfection Reagent	Thermo Fisher Scientific	STEM00001
Trypsin-EDTA (0.05%)	Thermo Fisher Scientific	25300054
Opti-MEM	Thermo Fisher Scientific	31985070
Puromycin	Millipore Sigma	540411
N2 supplement	Thermo Fisher Scientific	17502048
Insulin	Millipore Sigma	I9278
Doxycycline	Millipore Sigma	D9891
Neurobasal Medium	Thermo Fisher Scientific	21103049
Glutamax Supplement	Thermo Fisher Scientific	35050061
B27 supplement	Thermo Fisher Scientific	17504044
KnockOut Serum Replacement (KSR)	Thermo Fisher Scientific	10828028
KnockOut DMEM/F-12	Thermo Fisher Scientific	12660012
LDN193189	Bio-techne	6053
SB431542	Bio-techne	1614

(*Continued on next page*)

Continued

REAGENT or RESOURCE	SOURCE	IDENTIFIER
DAPT	Bio-techne	2634
CHIR99021	Bio-techne	4423
SU5402	Bio-techne	3300
BDNF	Bio-techne	248-BDB
GDNF	Bio-techne	212-GD
NGF	Bio-techne	256-GF
CytoView MEA 48 plates	Axion Biosystems	M768-tMEA-48B
Fetal Bovine Serum (FBS)	Thermo Fisher Scientific	MT35010CV
Ara-C	Millipore Sigma	C6645
TRIzol Reagent	Thermo Fisher Scientific	15596026
Histamine	Sigma-Aldrich	H7250
hIL-31	Peprotech	200-31
hIL-13	R&D Systems	213-ILB
(E)-capsaicin	Tocris Bioscience	0462
ABT-317	AbbVie	N/A
HBSS	Thermo Fisher Scientific	14175103
HEPES	Thermo Fisher Scientific	15630130
Papain	Worthington Biochemical Corporation	LS003126
Calcium chloride	Millipore Sigma	C1016
**Critical commercial assays**		
SuperScript IV First-Strand Synthesis System	Thermo Fisher Scientific	18091050
SYBR Green Master Mix for qPCR	Thermo Fisher Scientific	A25742
Chromium GEM-X Single Cell 3′ Kit	10× Genomics	CG000731
**Experimental models: Cell lines**		
WTC11	Coriell	UCSFi001-A; RRID:CVCL_Y803
KOLF2.1J	Jackson Laboratory	WTSIi018-B-12; RRID:CVCL_B5P3
HEK/293T	ATCC	HEK293T; RRID:CVCL_0063
**Recombinant DNA**		
pMDLg/pRRE	Addgene	12251
RSV-Rev	Addgene	12253
pCMV-VSV-G	Addgene	8454
pFUW-tetO-RUNX1	Addgene	139821
Teto-BRN3A	Addgene	62221
Super PiggyBac Transposase	System Biosciences	PB210PA-1
**Oligonucleotides**		

(*Continued on next page*)

Continued

REAGENT or RESOURCE	SOURCE	IDENTIFIER
GAPDH<br>Fw: 5′-AGCCACATCGCTCAGACAC-3′<br>Rev: 5′-CCCAATACGACCAAATCC-3′	This paper	N/A
MAP2	PrimerBank	87578393c1
PRPH	PrimerBank	66932907c1
TRPV1	PrimerBank	117306163c1
HRH1	PrimerBank	149158708c1
NPPB	PrimerBank	83700236c1
JAK1	PrimerBank	102469033c1
P2RX3	PrimerBank	332078493c1
RET	PrimerBank	126273513c1
SST	PrimerBank	71979669c1
**Software and algorithms**		
SnapGene	Snapgene.com	RRID:SCR_015052
Fiji	Open-source	RRID: SCR_002285
Adobe Illustrator	Adobe	RRID: SCR_010279
FastQC v0.12	Babraham Bioinformatics	RRID: SCR_014583
Trim Galore v0.6.10	Babraham Bioinformatics	RRID: SCR_011847
STAR aligner v2.7.11	GitHub	RRID: SCR_004463
DESeq2 R package v1.46.0	Bioconductor	RRID: SCR_015687
Kallisto v0.52.0	GitHub	RRID: SCR_016582
Cellranger V8.0.0	10× Genomics	RRID: SCR_023221
Metascape	Metascape	RRID: SCR_016620
Seurat (v4.9.9)	GitHub	RRID: SCR_016341
UCell	Bioconductor	RRID:SCR_027109
Neural Metric Tool	Axion Biosystems	N/A
QuPath (v0.6.0)	GitHub	RRID:SCR_018257
**Antibodies**		
anti-TUBB3	Biolegend	801201; RRID: AB_2313773
anti-MAP2	Abcam	AB5392; RRID: AB_2138153,
anti-TRPV1	Almone Lab	ACC-030; RRID: AB_2313819
anti-TRPA1	Novus Bioligicals	NB110-40763SS; RRID: AB_715124
anti-Trka	Almone Lab	ANT-018; RRID:AB_10658910
anti-IL31RA	Abcam	AB113498; RRID:AB_10860525
anti-OSMR	LSBio	LS-B11477-50; RRID: AB_3750299
anti-RET	Novus biologicals	AF1485; RRID: AB_354820

(*Continued on next page*)

Continued		
**REAGENT or RESOURCE**	**SOURCE**	**IDENTIFIER**
anti-HuNu	Novus Bioligicals	NBP3-13912; RRID:AB_3588766
anti-PRPH	Millipore	AB1530; RRID: AB_90725
Donkey anti-Chicken IgY, Alexa Fluor 488	Thermo Fisher Scientific	Cat#A-78948; RRID: AB_2921070
Donkey anti-Rabbit IgG, Alexa Fluor 555	Thermo Fisher Scientific	Cat#A-31572; RRID: AB_162543
Donkey anti-Rat IgG, Alexa Fluor 488	Thermo Fisher Scientific	Cat#A-21208; RRID: AB_141709
Donkey anti-Mouse IgG, Alexa Fluor 488	Thermo Fisher Scientific	Cat#A-21202; RRID: AB_141607
Donkey anti-Goat IgG, Alexa Fluor 555	Thermo Fisher Scientific	Cat#A-32816; RRID: AB_2762839
DAPI	Thermo Fisher Scientific	Cat# 62248
**Experimental models: Organisms/strains**		
Mouse: CD-1 IGS	Charles River Laboratories	Strain Code: 022
**Other**		
Stem Cell Engineering Core	Icahn School of Medicine at Mount Sinai	RRID:SCR_027503
Ts2R-FL inverted microscope	Nikon Instruments Inc	N/A
DMI4000 B inverted microscope	Leica Microsystems, GmbH	N/A
Axion Biosystems Maestro Pro System	Axion	RRID:SCR_026654
QuantStudio 5 Real-Time PCR System	Applied Biosystems	RRID:SCR_020240
Singulator 100 System	S2 Genomics	RRID:SCR_018155
**Deposited data**		
Raw and analyzed data	This paper	GEO: GSE329269
Human reference genome NCBI build 38, GRCh38	Genome Reference Consortium	http://www.ncbi.nlm.nih.gov/projects/genome/assembly/grc/human/

METHOD DETAILS

iPSCs maintenance

WTC11 and KOLF2.1J were cultured in 6-well plates coated with 1 mL of Geltrex (diluted 1:250 in DMEM/F12) and maintained in StemFlex. The medium was replaced every two days. When cultures reached ~80% confluency, cells were gently detached by incubation with 0.5 mM EDTA in DPBS for 8 min at room temperature. Differentiation was assessed morphologically under an optical microscope before passaging. Detached cells were resuspended in iPSC medium supplemented with 50nM Chroman 1 and seeded onto a new plate. The following day, the medium was replaced with fresh iPSC medium, and this process was repeated to maintain the iPSC culture.

Experiments were performed using cells derived from characterized master or working cell banks between passages 20–40, and cells were maintained for no more than 10 additional passages prior to differentiation and downstream experiments.

Cell line quality control

WTC11 and KOLF2.1J iPSC lines were routinely tested for mycoplasma contamination using the luminescence-based MycoAlert Mycoplasma Detection Kit (Lonza) and were confirmed negative prior to experimentation. Cell line identity and was verified by STR profiling and SNP-array matching to the original donor material/source cell line. No additional sterility (bacteriostasis/fungistasis) testing was performed.

Plasmids

The following plasmids were obtained from Addgene and used in this study: pMDLg/pRRE (Plasmid #12251) (Dull et al., 1998), RSV-Rev (Plasmid #12253) (Dull et al., 1998), pCMV-VSV-G (Plasmid #8454) (Stewart et al., 2003), pFUW-tetO-RUNX1 (Plasmid #139821) (Rosa et al., 2018), Teto-BRN3A (Plasmid #62221) (Blanchard et al., 2015), Super PiggyBac Transposase. TetO-FUW-NGN2-Puro, TetO-FUW-NGN1-Puro, TetO-ISL1, and TRE3G-BI-NGN1-ISL1-Puro were cloned and available upon request.

Lentivirus packaging

HEK/293T cells were cultured in MEF medium, prepared with 440 mL DMEM, 50 mL calf serum, 5 mL sodium pyruvate, 5 mL Non-Essential Amino Acids, and 4 μL of 2-Mercaptoethanol (14.3M). On the previous day of transfection 10 cm plates were coated with 5 mL of 15 μg/mL polyornithine in Dulbecco's phosphate buffered saline (DPBS), incubating at 37°C for 1 h, and washing with PBS twice. 7×10^6 cells of HEK/293T cells, dissociated using 0.05% Trypsin-EDTA, were seeded in 6 mL MEF media per plate. After 24 h, the medium was changed to fresh MEF media and cells were transfected using a polyethylenimine (PEI)-based method. DNA (20 μg total: 10 μg of lentivirus plasmid, 5 μg of pMDLg/pRRE, 2.5 μg of RSV-Rev, 2.5 μg of pCMV-VSV-G) was mixed with 500 μL DMEM (Tube A), while 60 μL of 1 mg/mL PEI 25K in DPBS was mixed with 500 μL DMEM (Tube B). Tubes A and B were combined and incubated for 20 min at RT, then added dropwise to the cells (1mL per plate). 6 h post-transfection, cells were washed with PBS and prewarmed MEF media was added. Supernatants were collected at 30 and 46 h post-transfection, filtered through a 0.45 μm filter. The virus was concentrated by ultracentrifugation at 21,000 rpm for 2 h at 4°C. The resulting pellet was resuspended in DMEM in a volume 100 times smaller overnight. Then same amount of 1M sucrose in DMEM was added and stored at −80°C after being aliquoted.

Generation of iPSC lines with PiggyBac-integrated transgenes

WTC11 or KOLF2.1J were seeded in 3cm dish in StemFlex medium supplemented with Chroman1 and cultured overnight. On day 1, cells were transfected with one PiggyBac plasmid: TRE3G-BI-NGN1-ISL1-Puro and Super PiggyBac Transposase—using Lipofectamine. Transfection complexes were prepared by mixing Lipofectamine and plasmid DNA at a ratio of 1:5 (and maintaining a transposase:vector ratio of 1:2) in Opti-MEM; after a 10 min incubation at RT, 250 μL of the mixture was added dropwise to cells pre-equilibrated in 2 mL warm Opti-MEM I reduced serum medium. Following a 4 hour-incubation at 37°C, 2 mL of warm StemFlex medium was added, and the cells were cultured overnight. On day 2, the medium was aspirated and replaced with 1.5 mL of fresh warm StemFlex. Selection was initiated on day 3 by switching to StemFlex medium supplemented with 2 μg/mL puromycin. The selection was maintained for 4–6 days with medium changes every other day. Cells were then passaged once in StemFlex containing both puromycin and Chroman1 to ensure uniform exposure to puromycin and eliminate false-positive cells.

Neural induction using forced expression of TFs with lentivirus on iPSC (one-step)

On day −1 WTC11 or KOLF2.1J cells in 6-well plate with PSC medium were detached by being incubated with Accutase for 5 min at 37°C and resuspended with PSC medium containing Chroman1 and lentivirus carrying TetO-FUW-NGN2-Puro or TetO-FUW-NGN1-Puro for transfection. Lentivirus was used at a volume of 0.5 μL per well in a 6-well plate. The virus was prepared alongside a TetO-FUW-GFP construct, which consistently yielded >70% GFP-positive cells under the same infection conditions. Based on this, the MOI was estimated to be approximately 10. The cells were seeded in each well of 6-well plates, coated with Geltrex in DMEM/F12. From day 0, the medium was switched to N3 medium, prepared mixing the followings; 490 mL of DMEM/F12, 5 mL of N2 supplement, 5 mL of NEAA, and 10 mg of Insulin. And 2μg/mL doxycycline was added from day 0–6 to activate NGN1 or NGN2 expression. 2μg/mL puromycin was used for selection on day 1 and 2. On day 7, cells were detached by being incubated with Accutase for 20 min at 37°C and resuspended in Neurobasal-B27 medium containing Chroman1. Neurobasal-B27 medium was prepared by mixing the followings; 485 mL of Neurobasal, 5mL of Glutamax, 10 mL of B27. The cells were seeded in each well of 6-well plates, coated with Geltrex. After that the cells were maintained in Neurobasal-B27 medium until the day of the analysis.

Neural induction using forced expression of TFs with lentivirus or PiggyBac on NCCs (2-step)

On day −1 WTC11 cells or KOLF2.1J cells were detached by being incubated with Accutase for 5 min at 37°C and resuspended with Stemflex containing Chroman1. The cells were seeded in each well of 6-well plates, coated with Geltrex. On day 0, the medium is changed to KSR medium; 192.5 mL of Knockout-DMEM-F12, 50 mL of KnockOut Serum Replacement (KSR), 2.5 mL of Glutamax, 5mL of NEAA, and 2 μL of 14.3M 2-Mercaptoethanol. The medium was gradually

transitioned from KSR to Neurobasal-B27 from day 0 to day 11. From day 0–7, 100 μM of LDN193189, and 10 μM of SB431542 and from day 4 to day 11, 10 μM of DAPT, 3 μM of CHIR99021, and 10 μM of SU5402 are added to the culture medium. On day 11, the cells were detached by being incubated with Accutase for 20 min at 37°C and resuspended with Neurobasal-B27 medium containing Chroman1. The cells were seeded in each well of 6-well plates, coated with Geltrex. Lentivirus carrying TetO-FUW-NGN1-Puro or TetO-FUW-NGN2-Puro, TetO-ISL1, TetO-BRN3A, or pFUW-tetO-RUNX1 were added as described in each experiment. Doxycycline was added from day 12 to day 18 induce gene expression. 2μg/mL puromycin was added on day 13 for selection for lentivirus-transfected cells. 25 ng/mL of BDNF and 25 ng/mL of GDNF were added from day11–18. For RNA-extraction, 25 ng/mL of NGF was added instead of BDNF and GDNF from day 18 to the day of analysis, day 21 (for the experiment for Figure S2C, NGF was not added and BDNF and GDNF were kept added in one group). For immunofluorescence staining (IF) and MEA, on day 18, the cells were detached with Accutase for 20 min at 37°C and resuspended with Neurobasal-B27 medium containing Chroman1. 100,000 cells were seeded with 20,000 mouse glia cells onto coverslip coated with Geltrex in 24 well plate for IF and 24 or CytoView MEA 48 plates coated with Geltrex for MEA. From the day after this replate, the cells were cultured in Neurobasal-B27 medium with 25 ng/mL of NGF and 2% FBS to the day of analysis (for the experiment for Figures 4B, S2B, and S2C, NGF was not added and BDNF and GDNF were kept added in one group). From day 28, 4 μM of Ara-C was added.

RNA extraction and quantitative real-time PCR

Total RNA was extracted using TRIzol. Briefly, samples were lysed and homogenized in TRIzol following the manufacturer's protocol. After chloroform addition, the lysates were centrifuged at 12,000 × g for 5 min at 4°C to achieve phase separation. The aqueous phase containing RNA was carefully transferred. RNA was precipitated with isopropanol, washed with 75% ethanol, and resuspended in RNase-free water. For cDNA synthesis, 200 ng to 1 μg of RNA was reverse transcribed using SuperScript IV First-Strand Synthesis System following the manufacturer's instructions. The resulting cDNA was stored at −20°C until use. Quantitative real-time PCR was conducted using SYBR master mix. The reactions were carried out in 384-well plates on an Applied Biosystems QuantStudio 5 instrument. The sequences of primers are listed in the key resources table. For each experiment, *GAPDH* was utilized as housekeeping control. The RT-qPCR data were analyzed using the comparative CT method. Gene expression levels were normalized to *GAPDH*, and relative expression was determined using the level in iPSCs (normalized to *GAPDH*) as the reference value (set to 1) for group comparisons.

Bulk RNA-seq

For RNA library preparation Poly(A) selection was used to enrich for mRNA, followed by sequencing on an Illumina platform with a 2 × 150 bp configuration, aiming for 50 million paired-end reads per sample. Quality control was performed using FastQC, ensuring that all samples met the thresholds for basic statistics, per-base sequence quality, and per-sequence quality scores. Adapter sequences and low-quality bases were removed using Trim Galore. The cleaned reads were aligned to the GRCh38 genome assembly using STAR (Dobin et al., 2013) with the GENCODE v29 annotation to generate BAM files and gene count data. Transcript abundance was quantified using Kallisto (Bray et al., 2016) with GENCODE v29 annotation and the GRCh38 genome assembly. To calculate TPM values, Kallisto abundance estimates were used and aggregated to the gene level using transcript-to-gene mapping derived from GENCODE annotations. Log-transformed TPM values ($\log_{10}$(TPM +1)) were computed for normalization and subsequently standardized to Z-scores for comparative visualization. For the heatmap of selected genes, the standardized (Z-scores) log-transformed TPM values were used to visualize the expression patterns of predefined genes of interest across samples. This provided a focused view of key gene expression dynamics. For clustering to assess overall similarity between groups, DESeq2 (Love et al., 2014) was employed to identify differentially expressed genes (DEGs) via a likelihood ratio test (LRT). Significant DEGs (p-adj <0.05) were used for hierarchical clustering and heatmap visualization. Pairwise differential expression analysis between two groups (N1, N2 and N1+I vs. N1+B, N1+R, N1+I vs. N1+B, or N1+I vs. NI + R) was performed using DESeq2, with an adjusted *p*-value threshold of 0.05. Volcano plots were generated using the DESeq2 output. Ontology enrichment and tissue-specificity analyses of the DEGs were performed with Metascape, using TOP 300 DEGs in each group.

Single-nucleus RNA sequencing (snRNA-seq)

iPSCs (day 0) and iPruriceptors (day 21) cultured without mouse glia were collected using Accutase, washed with cold 2.5% BSA, centrifuged at 400 g at 4°C for 5 min, snap-frozen, and stored at −80°C until nuclei isolation. Frozen samples were processed using the Singulator 100 System to generate single-nucleus suspensions, followed by library preparation

with the Chromium GEM-X Single Cell 3′ Kit according to the manufacturer's protocol. Libraries were sequenced on the Illumina platform NovaSeq X Plus, and gene-count matrices were generated using CellRanger (Satija et al., 2015). In total, 24,808 iPSC nuclei and 22,005 iPruriceptor nuclei were sequenced. Downstream analyses were performed using Seurat (Hao et al., 2021). Low-quality nuclei were filtered (500 < nFeature_RNA <6,000; percent.mt < 5%), normalized, and scaled. Cell-cycle scores were computed using curated S- and G2/M-phase gene sets and regressed out during scaling to minimize proliferation-driven variation (Tirosh et al., 2016). Dimensionality reduction was performed using PCA and UMAP with graph-based clustering. Cell identity was evaluated using UCell gene-program scoring with curated subtype signatures (Andreatta and Carmona, 2021), and marker expression across clusters was visualized using Seurat DotPlot.

Multielectrode array (MEA)

Using the Maestro Pro, the activity of the cells was typically measured for 10 min in a 37°C and 5% CO_2 atmosphere. Recordings were taken at a sample rate of 12.5 kHz/channel with a Butterworth band-pass filter (200 Hz–3000 Hz). Positive and negative deflecting spikes were detected using an adaptive threshold method set at 6× standard deviation of the noise floor. Stimulation assays were performed primarily on day 42 of differentiation. For experiments shown in Figure 4, individual wells were exposed to a single stimulus (i.e., no matched or sequential stimulation within the same well), and different stimuli were assessed in independent wells across experiments. When the same culture batch was reused for separate experiments, wells were rinsed twice with PBS immediately after recording and allowed to recover for at least 3 days in fresh medium before the next stimulation. For stimulation experiments, after baseline measurements, a 10-fold higher concentration of reagent than the final concentration was quickly added to the existing culture solution at 1/9 of the volume and measured for 10 min after addition. Stock solutions were stored in PBS or DMSO at even higher concentrations, with PBS and DMSO being less than 0.1% of the total solution at the time of measurement. The following substances were used for stimulation: 1 mM of Histamine, 300 ng/mL of IL-31, 300 ng/mL of IL-13, 10 μM of (E)-capsaicin. For the JAK1 inhibition experiment, ABT-317 (provided by AbbVie) was added at a final concentration of 1 μM and incubated at 37°C for 1 h, followed by the addition of hIL-31 at a final concentration of 300 ng/mL. 10 min before and 10 min after the addition of hIL-31 were recorded. The analysis was conducted using Neural Metric Tool. Mean firing ratio at 10 min post-administration, normalized to 1 for the Mean Firing Ratio at 10 min before administration, was used for comparison between groups. Each data point represents one well. Wells exhibiting minimal or absent spontaneous activity (indicative of insufficient neuronal network formation) were excluded prior to statistical analysis. Outliers were identified and excluded using the inter-quartile-range (IQR) method: any well whose mean firing rate fell below $Q1 - 1.5 \times IQR$ or exceeded $Q3 + 1.5 \times IQR$ was removed.

Immunofluorescence staining

Cells were washed with PBS^{+++} (PBS supplemented with 4 g/100 mL sucrose, 50 μL 0.5M $CaCl_2$, and 50 μL 1M $MgCl_2$), and fixed with 4% paraformaldehyde (PFA) containing 4% sucrose for 5 min at RT. After washing three times with PBS^{+++}, cells were incubated with 0.2% Triton X- for 5-10 min at room temperature to permeabilize the membranes. Blocking was performed with blocking buffer (PBS containing 4% BSA, 1% FBS, and NaN_3) for 2 h at RT. The cells were then incubated overnight at 4°C with the following primary antibodies diluted in blocking buffer: anti-TUBB3 (1:1000), anti-MAP2 (1:20000), anti-TRPV1 (1:200), anti-TRPA1 (1:1000), anti-Trka (1:200), anti-IL31RA (1:200), anti-OSMR (1:200), anti-RET (1:200), anti-HuNu (1:100), anti-PRPH (1:200). After incubation, cells were washed three times with PBS and incubated for 1 h at RT in the dark with secondary antibodies diluted 1:500 in blocking buffer. Cells were then washed three times with PBS, and 0.2ng/mL of DAPI was added in the final wash for nuclear staining. Coverslips were mounted using ProLong Gold Antifade Mountant with DNA Stain DAPI, and imaging was performed using a DMI4000 B Automated Inverted Microscope. Fluorescence images were analyzed using QuPath (Bankhead et al., 2017), an open-source software for digital pathology. The positive-cell-detection tool was applied using either the DAPI or HuNu nuclear stain to segment individual cells. These detected cells served as the basis for further quantification of fluorescence intensity in additional channels corresponding to neuronal and receptor markers: MAP2, PRPH, TRPA1, RET, TRPV1, IL31RA, OSMR, and TrkA. Marker expressions were quantified within the nuclear or perinuclear region depending on subcellular localization, and thresholds were adjusted to account for background fluorescence and channel-specific signal ranges.

Mice

CD1 mice were housed in specific-pathogen-free (SPF) conditions and environmentally controlled animal facilities with a 12-h light-dark cycle and were given unrestricted access to food and water. All animal protocols and experiments were

approved by the Institutional Animal Care and Use Committee (IACUC) of ISMMS. All experiments were performed following strict IACUC guidelines.

Mouse glia cell preparation

CD1 pups (postnatal P1-P3) were euthanized via hypothermia-induced anesthesia followed by humane euthanasia on ice, in accordance with institutional animal care guidelines. Cortices were dissected in ice-cold HBSS with 10mM HEPES, and meninges were removed. The tissue was then digested with 80 μL of papain, activated with 0.5 μM $CaCl_2$ and 1 μM EDTA, at 37°C for 15 min. Following digestion, cells were triturated and filtered through a 70 μm strainer before centrifugation at 1000 rpm for 5 min. The resulting cell pellet was resuspended in MEF medium. Cells were seeded in a 10 cm dish, with the medium changed after 24 h. After 5–6 days in culture, cells were detached, and cryopreserved in Bambanker until use.

Stem Cell Reports, Volume *21*

Supplemental Information

Induction of human pruriceptors from pluripotent stem cells via transcription factors

Hisato Iriki, Ruiqi Hu, Xu Li, Erdene Baljinnyam, Carina Habich, Ichiro Imanishi, Loan N. Miller, Kavya Chegireddy, Laraib Iqbal Malik, Daniel Yassky, Raphael Kübler, Aaron Ver Heul, Kathleen M. Smith, Peter Reinhardt, Eric R. Goedken, Brian S. Kim, and Samuele G. Marro

Supplemental Material

Supplemental Figures

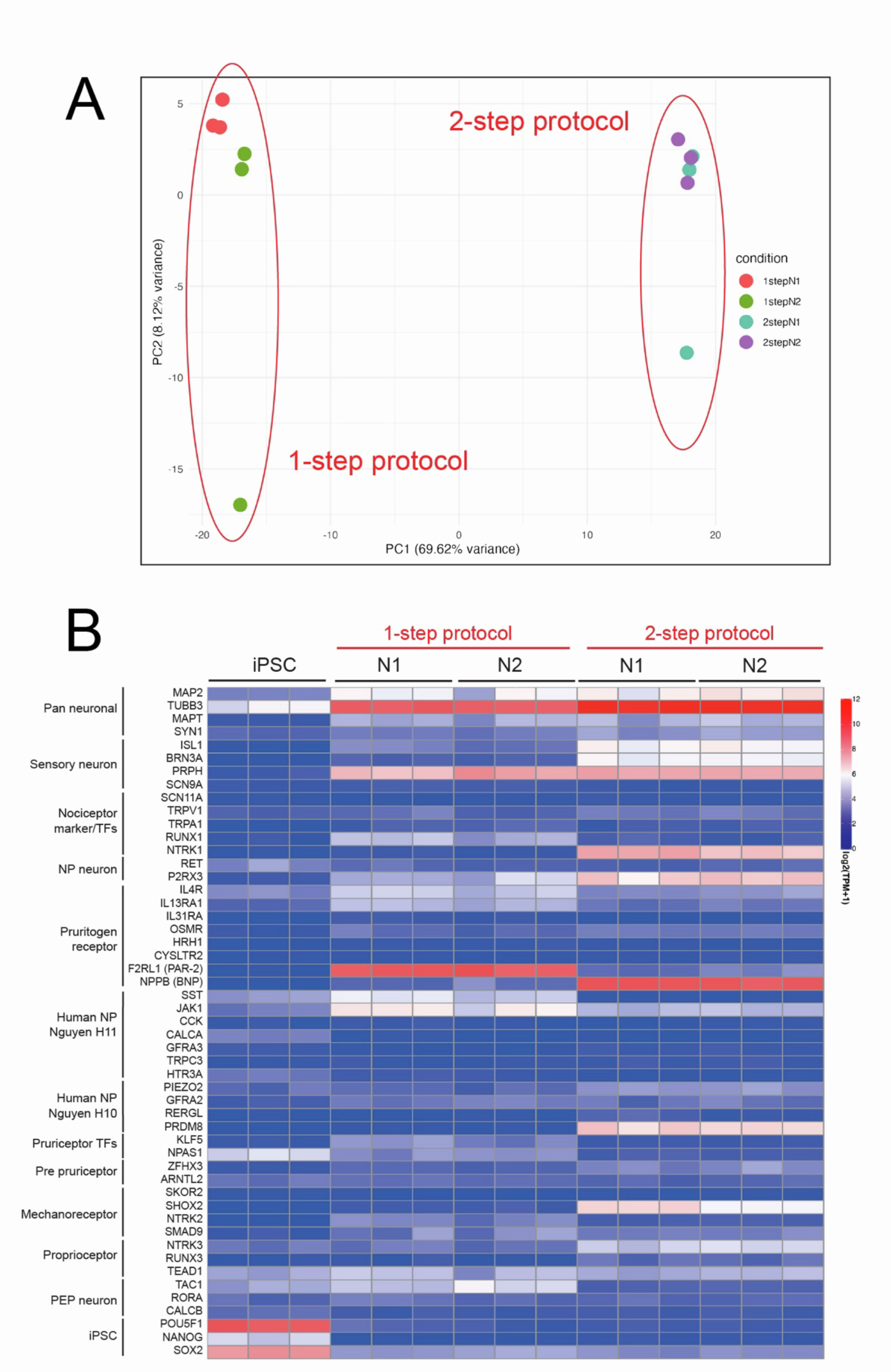

Figure S1. NGN1 expression in NCCs shows a tendency to enhance pruriceptor marker induction. Related to Figure 1.

(A) PCA of transcriptomic profiles. Each point represents a sample colored by condition: 1stepN1 (red), 1stepN2 (green), 2stepN1 (cyan), and 2stepN2 (purple). PC1 and PC2 explain 69.62% and 8.12% of the variance, respectively.

(B) Heatmap analysis of samples obtained on protocol day 21 using either the 1-step or 2-step protocol with NGN1 or NGN2 expression (1-stepN1, 1-stepN2, 2-stepN1, and 2-stepN2), along with iPSCs, showing the expression of the indicated markers. Transcript expression values (TPM) were log2-transformed, n=3. The analyzed data is same as the one analyzed in Figure 1C.

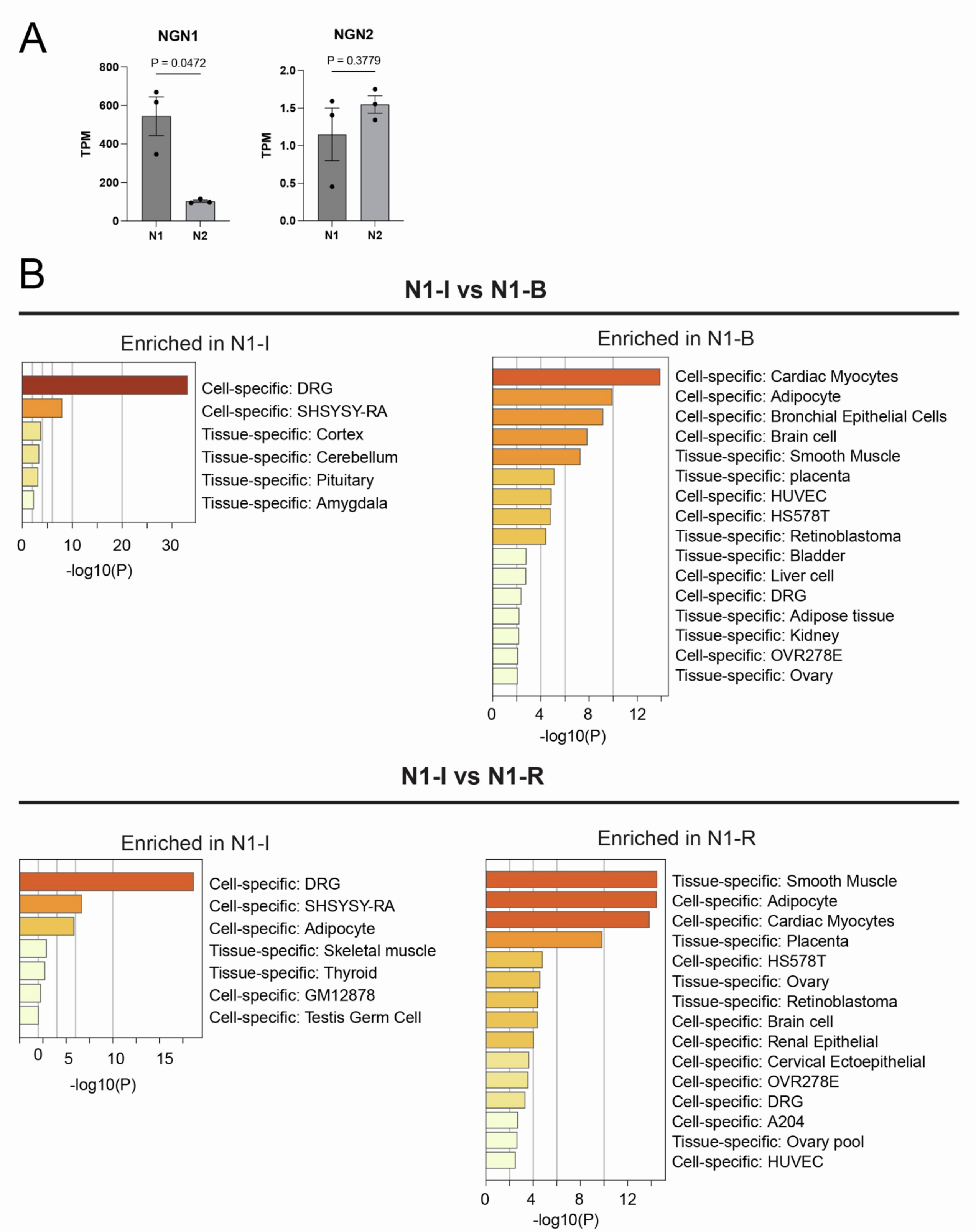

A
NGN1
P = 0.0472
TPM
800
600
400
200
0
N1
N2
NGN2
P = 0.3779
TPM
2.0
1.5
1.0
0.5
0.0
N1
N2
B
N1-I vs N1-B
Enriched in N1-I
Cell-specific: DRG
Cell-specific: SHSYSY-RA
Tissue-specific: Cortex
Tissue-specific: Cerebellum
Tissue-specific: Pituitary
Tissue-specific: Amygdala
0
10
20
30
-log10(P)
Enriched in N1-B
Cell-specific: Cardiac Myocytes
Cell-specific: Adipocyte
Cell-specific: Bronchial Epithelial Cells
Cell-specific: Brain cell
Tissue-specific: Smooth Muscle
Tissue-specific: placenta
Cell-specific: HUVEC
Cell-specific: HS578T
Tissue-specific: Retinoblastoma
Tissue-specific: Bladder
Cell-specific: Liver cell
Cell-specific: DRG
Tissue-specific: Adipose tissue
Tissue-specific: Kidney
Cell-specific: OVR278E
Tissue-specific: Ovary
0
4
8
12
-log10(P)
N1-I vs N1-R
Enriched in N1-I
Cell-specific: DRG
Cell-specific: SHSYSY-RA
Cell-specific: Adipocyte
Tissue-specific: Skeletal muscle
Tissue-specific: Thyroid
Cell-specific: GM12878
Cell-specific: Testis Germ Cell
0
5
10
15
-log10(P)
Enriched in N1-R
Tissue-specific: Smooth Muscle
Cell-specific: Adipocyte
Cell-specific: Cardiac Myocytes
Tissue-specific: Placenta
Cell-specific: HS578T
Tissue-specific: Ovary
Tissue-specific: Retinoblastoma
Cell-specific: Brain cell
Cell-specific: Renal Epithelial
Cell-specific: Cervical Ectoepithelial
Cell-specific: OVR278E
Cell-specific: DRG
Cell-specific: A204
Tissue-specific: Ovary pool
Cell-specific: HUVEC
0
4
8
12
-log10(P)

Figure S2. ISL1 contributes to differentiation into DRG neurons when co-expressed with NGN1. Related to Figure 2.

(A) Bar graphs showing TPM of NGN1 and NGN2 in N1 and N2. Each bar represents the mean ± SEM, and individual data points are shown. Statistical comparisons between the two groups were performed using an unpaired t-test with Welch's correction.

(B) Metascape tissue-specific enrichment of the top 300 DEGs: upper panels show 2-step N1-I vs N1-B and lower panels 2-step N1-I vs N1-R; within each comparison, left bars represent tissues/cell types enriched among genes up-regulated in N1-I and right bars those enriched in N1-B or N1-R, with bar length indicating $-\log_{10}$ P.

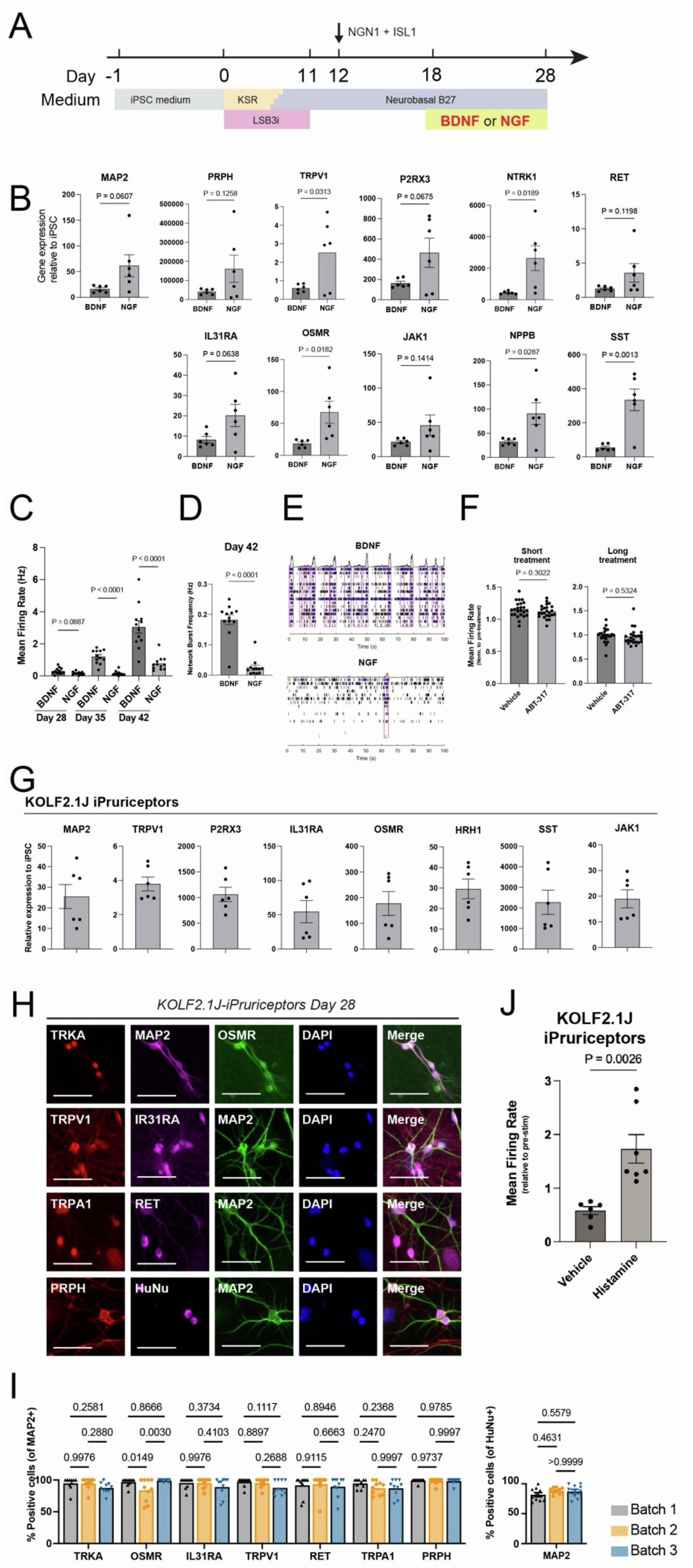
A
NGN1 + ISL1
Day -1 0 11 12 18 28
Medium
iPSC medium
KSR
Neurobasal B27
LSB3i
BDNF or NGF
B
MAP2
PRPH
TRPV1
P2RX3
NTRK1
RET
P = 0.0607
P = 0.1258
P = 0.0313
P = 0.0675
P = 0.0189
P = 0.1198
Gene expression relative to iPSC
BDNF NGF
IL31RA
OSMR
JAK1
NPPB
SST
P = 0.0638
P = 0.0182
P = 0.1414
P = 0.0287
P = 0.0013
C
Mean Firing Rate (Hz)
P = 0.0887
P < 0.0001
P < 0.0001
Day 28
Day 35
Day 42
D
Day 42
P < 0.0001
Network Burst Frequency (Hz)
E
BDNF
NGF
Time (s)
F
Short treatment
Long treatment
P = 0.3022
P = 0.5324
Mean Firing Rate (Norm. to pre-treatment)
Vehicle
ABT-317
G
KOLF2.1J iPruriceptors
MAP2
TRPV1
P2RX3
IL31RA
OSMR
HRH1
SST
JAK1
Relative expression to iPSC
H
KOLF2.1J-iPruriceptors Day 28
TRKA
MAP2
OSMR
DAPI
Merge
TRPV1
IR31RA
TRPA1
RET
PRPH
HuNu
J
KOLF2.1J iPruriceptors
P = 0.0026
Mean Firing Rate (relative to pre-stim)
Vehicle
Histamine
I
0.2581
0.8666
0.3734
0.1117
0.8946
0.2368
0.9785
0.2880
0.0030
0.4103
0.8897
0.6663
0.2470
0.9997
0.9976
0.0149
0.9976
0.2688
0.9115
0.9997
0.9737
% Positive cells (of MAP2+)
TRKA
OSMR
IL31RA
TRPV1
RET
TRPA1
PRPH
0.5579
0.4631
>0.9999
% Positive cells (of HuNu+)
MAP2
Batch 1
Batch 2
Batch 3

Figure S4. iPruriceptors functionally respond to pruritogenic stimuli and JAK1 inhibition. Related to Figure 4.

(A) Schematic showing co-expression of NGN1 and ISL1 in NCCs induced by LSB3i via lentiviral transduction, followed by culture with BDNF or NGF from day 18 to day 28, the time point of quantitative PCR analysis.

(B) Quantitative PCR analysis of indicated markers on day 28 in the protocols under different neurotrophic conditions (BDNF vs NGF) after day 18. Relative expression levels were normalized to control iPSC levels. Data are presented as mean ± SEM, with individual data points representing independent biological replicates. Statistical comparisons were conducted using an unpaired t-test.

(C, D) MEA analysis showing maturation of neuronal activity over time under different neurotrophic conditions (BDNF vs NGF) after day 18. Mean firing rates were measured on days 28, 35, and 42, and network burst frequency was measured on day 42. The values represent the mean firing rate on (C) and network burst frequency on (D). Data are presented as mean ± SEM. Each dot represents one well. Representative data from three independent experiments (n = 12). Statistical comparisons between the two groups were performed using an unpaired t-test with Welch's correction.

(E) MEA raster plots showing neuronal spiking activity of neuronal activity with displaying network burst enclosed in purple box on day 42 under different neurotrophic conditions (BDNF vs NGF) after day 18. Each vertical line represents an action potential recorded from a single electrode.

(F) MEA analysis showing firing rates after short (30 sec) or long (60 min) treatment of JAK1 inhibitor, ABT-317, or vehicle treatment. Data are presented as mean ± SEM. Each dot represents one well. Pooled data from two independent experiments (n = 12 each).

(G) Quantitative PCR analysis of indicated markers on day 21 in the protocols using KOLF2.1J cells. Relative expression levels were normalized to control iPSC levels. Data are presented as mean ± SEM.

(H) Immunofluorescence staining of indicated markers on the induced neurons under iPruriceptor protocol from KOLF2.1J on day 28. Scale bars indicate 50μm.

(I) Bar graph showing the proportion of induced neurons under iPruriceptor protocol expressing the indicated markers on day 28. Data are presented as mean ± SEM across three independent batches. Each dot represents one field of view. Statistical comparisons between the batches were performed using a two-way ANOVA test.

(J) MEA analysis showing neuronal responses to histamine in induced neurons, using KOLF2.1J cell line, on day 42 from the iPruriceptor protocol maintained with NGF after day 18. Representative data from two independent experiments (n = 6 or 7). Values represent mean firing rates normalized to the average baseline across wells per condition. Each dot represents one well. Statistical comparisons between the two groups were performed using an unpaired t-test with Welch's correction.

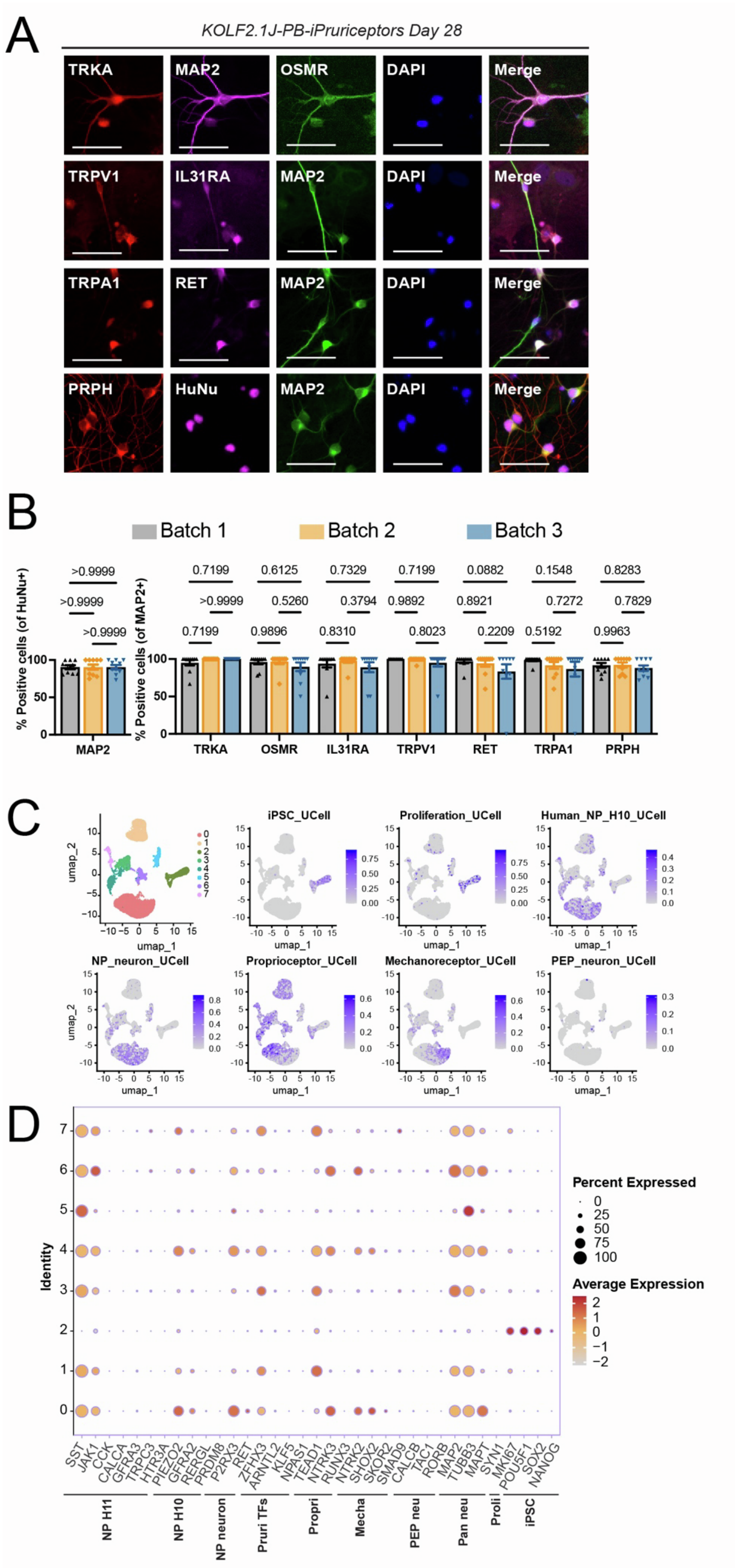
A
KOLF2.1J-PB-iPruriceptors Day 28
TRKA
MAP2
OSMR
DAPI
Merge
TRPV1
IL31RA
MAP2
DAPI
Merge
TRPA1
RET
MAP2
DAPI
Merge
PRPH
HuNu
MAP2
DAPI
Merge
B
Batch 1
Batch 2
Batch 3
% Positive cells (of HuNu+)
% Positive cells (of MAP2+)
>0.9999
>0.9999
>0.9999
0.7199
0.6125
0.7329
0.7199
0.0882
0.1548
0.8283
>0.9999
0.5260
0.3794
0.9892
0.8921
0.7272
0.7829
0.7199
0.9896
0.8310
0.8023
0.2209
0.5192
0.9963
MAP2
TRKA
OSMR
IL31RA
TRPV1
RET
TRPA1
PRPH
C
umap_1
umap_2
iPSC_UCell
Proliferation_UCell
Human_NP_H10_UCell
NP_neuron_UCell
Proprioceptor_UCell
Mechanoreceptor_UCell
PEP_neuron_UCell
D
Identity
Percent Expressed
Average Expression
SST JAK1 CCK CALCA GFRA3 TRPC3 HTR3A PIEZO2 GFRA2 RERGL PRDM8 P2RX3 RET ZFHX3 ARNTL2 KLF5 NPAS1 TEAD1 NTRK3 RUNX3 NTRK2 SHOX2 SKOR2 SMAD9 CALCB TAC1 RORB MAP2 TUBB3 MAPT SYN1 MKI67 POU5F1 SOX2 NANOG
NP H11
NP H10
NP neuron
Pruri TFs
Propri
Mecha
PEP neu
Pan neu
Proli
iPSC

Figure S5. iPruriceptors generated with PiggyBac system reproduce robust functional pruriceptor induction. Related to Figure 5.

(A) Immunofluorescence staining of indicated markers on the induced neurons under iPruriceptor protocol with PiggyBac on day 28 from KOLF2.1J. Scale bars indicate 50µm. (B) Bar graph showing the proportion of induced neurons under iPruriceptor protocol with PiggyBac expressing the indicated markers on day 28 from KOLF2.1J. Data are presented as mean ± SEM across three independent batches. Each dot represents one field of view. Statistical comparisons between the batches were performed using a two-way ANOVA test.

(C) UMAP visualization of snRNA-seq clusters with overlaid UCell scores for curated gene signatures. These scores reveal enrichment of neuronal subtype signatures and pluripotency signatures in iPruriceptor and iPSC.

(D) DotPlot showing expression of representative marker genes across clusters. Dot size indicates the percentage of nuclei expressing each gene, and color intensity represents average expression level, supporting the assignment of pruriceptor subtypes and differentiation stage identities.

Supplemental Tables

Table S1. Differentially expressed genes (DEGs) identified between the N1/N2/N1-I group and the N1-B/N1-R group generated using the 2-step differentiation protocol.

Table S2. Differentially expressed genes (DEGs) identified between the N1-I condition and the N1-B or N1-R conditions generated using the 2-step differentiation protocol.
